# ZBED6 deficiency drives pathological cardiac hypertrophy through MEOX1–ERK1/2–dependent autophagy dysfunction

**DOI:** 10.1371/journal.pgen.1012329

**Published:** 2026-10-08

**Authors:** Xiao Zhang, Xiaopeng Liu, Ruirui Pan, Huan Liu, Junying Xiao, Xiangkai Leng, Hongmei Zhang, Dengke Pan, Shengzhong Duan, Jiangwei Wu

**Affiliations:** 1 Key Laboratory of Animal Genetics, Breeding and Reproduction of Shaanxi Province, College of Animal Science and Technology, Northwest A&F University, Yangling, Shaanxi, China; 2 Ultrasound in Cardiac Electrophysiology and Biomechanics Key Laboratory of Sichuan Province, Sichuan Provincial People’s Hospital, University of Electronic Science and Technology of China, Chengdu, China; 3 Clinical Immunology Translational Medicine Key Laboratory of Sichuan Province, Sichuan Academy of Medical Sciences & Sichuan Provincial People’s Hospital, Chengdu, Sichuan, China; 4 Stomatology Hospital, School of Stomatology, Zhejiang University School of Medicine, Zhejiang Provincial Clinical Research Center for Oral Diseases, Key Laboratory of Oral Biomedical Research of Zhejiang Province, Engineering Research Center of Oral Biomaterials and Devices of Zhejiang Province, Hangzhou, China; Iowa State University, UNITED STATES OF AMERICA

## Abstract

Pathological cardiac hypertrophy, a major risk factor for heart failure, is regulated by complex transcriptional networks. We previously identified the transcription factor ZBED6 as a key regulator for skeletal muscle hypertrophy, its role in the cardiac hypertrophy remains unexplored. Here, we report that ZBED6 is a key suppressor of pathological cardiac hypertrophy. ZBED6 is markedly reduced in myocardial tissues from patients with cardiac hypertrophy. ZBED6-deficient pigs exhibited cardiac hypertrophy with diastolic dysfunction. Integrative transcriptomic and ChIP-seq analyses identified MEOX1 as a direct negative target of ZBED6. Loss of ZBED6 upregulates MEOX1 transcription, which subsequently activated ERK1/2 signaling, leading to impaired autophagy-induced hypertrophy. Knockdown of MEOX1 reduced ERK1/2 phosphorylation and mitigated cardiomyocyte hypertrophy induced by ZBED6 deficiency. Similarly, pharmacological inhibition of ERK1/2 restored autophagy and alleviated hypertrophy in ZBED6-depleted cardiomyocytes. Collectively, these findings define a previously unrecognized ZBED6–MEOX1–ERK1/2 axis that critically regulates cardiac hypertrophy and represents a potential therapeutic target.

## Author summary

An enlarged heart may initially help the body cope with stress, but persistent enlargement can impair heart function and increase the risk of heart failure. We investigated whether a gene regulator called ZBED6 helps protect the heart from this harmful growth. We found that ZBED6 levels were reduced in heart tissue from people with cardiac hypertrophy. Pigs lacking ZBED6 developed enlarged hearts, thicker heart walls, enlarged heart muscle cells, and impaired relaxation, while their pumping ability remained largely preserved. Using pig and human heart cells, we found that loss of ZBED6 increased the activity of another gene, MEOX1. This change disrupted the cellular recycling process that removes damaged or unnecessary material. Reducing MEOX1 activity or blocking the downstream signal restored cellular recycling and limited heart-cell enlargement. Our findings identify ZBED6 as an important brake on harmful heart growth and reveal a previously unknown link between gene regulation, cellular recycling, and cardiac hypertrophy. This work may guide future studies seeking new approaches to prevent or treat pathological heart enlargement.

## Introduction

Pathological cardiac hypertrophy is tightly associated with cardiac dysfunction and represents a major risk factor for heart failure and sudden death [1]. Hypertrophic cardiomyopathy (HCM), a well-established form of pathological hypertrophy, often arises from genetically determined defects in sarcomeric proteins and is characterized by left ventricular hypertrophy, hypercontractility, and diastolic dysfunction [2,3]. Although mutations in sarcomeric genes such as the myosin heavy chain 7 (MYH7) gene [4–6], MYBPC3 and TNNT2 [4,7] are well-established genetic predisposing factors for inherited forms of cardiac hypertrophy, a substantial proportion of cardiac hypertrophy patients lack pathogenic variants in these genes, indicating that additional regulatory mechanisms contribute to disease development. Identifying these factors is essential for discovering new biomarkers and therapeutic targets.

Zinc finger BED-type containing 6 (ZBED6) is a highly conserved transcription factor originally identified through quantitative trait locus (QTL) analysis of muscle growth and fat deposition in European wild boars and Large White pigs, followed by oligonucleotide-capture and mass-spectrometry experiments [8,9]. ZBED6 acts as a transcriptional repressor of skeletal muscle and organ growth. We and others previously observed cardiac enlargement in ZBED6^-/-^ pigs and mice [10,11], suggesting an important role for ZBED6 in cardiac development. However, whether this cardiac enlargement represents physiological or pathological hypertrophy remains unclear, and the molecular mechanisms underlying ZBED6-mediated cardiac regulation have not been defined.

As a transcription factor, ZBED6 regulates the transcription of a series of downstream target genes by binding a specific DNA motif (GCTCG) [9]. IGF2 was the first identified ZBED6 target in skeletal muscle, where ZBED6 binding to its promoter suppresses IGF2 expression, regulating skeletal muscle growth [10,11]. We later identified DOCK3 as another direct ZBED6 target in porcine skeletal muscle during sepsis-induced muscle atrophy, highlighting the context dependence of ZBED6-mediated transcriptional regulation [12]. Mesenchyme Homeobox 1 (MEOX1) is a key regulator of pathological cardiac hypertrophy, with elevated expression observed in hypertrophic mouse and human hearts. Genetic deletion of *Meox1* alleviates pathological hypertrophy in mice, establishing a causal role for MEOX1 in cardiac hypertrophy [13,14]. Intriguingly, our preliminary sequence analysis revealed a putative ZBED6-binding motif in the promoter region of porcine *MEOX1*, suggesting a previously unrecognized regulatory relationship between ZBED6 and MEOX1 that might be crucial for cardiac hypertrophy.

Autophagy is essential for maintaining cardiomyocyte size and overall cardiac structure and function. While moderate autophagy is protective during early hypertrophic stress [15,16], chronic autophagy dysregulation, either excessive or impaired, can drive pathological cardiac hypertrophy [17,18]. Autophagy is governed by multiple pathways, including mTOR [18,19] and MAPK cascades [20]. The MAPK-ERK1/2 can either promote or inhibit autophagy depending on cellular context, a classic signal regulation in the autophagic process [21]. Notably, MAPK-ERK1/2 pathway is implicated in the development of cardiac hypertrophy [22–24]. Whether ZBED6 deficiency-induced cardiac hypertrophy in pigs through autophagy dysregulation remains unknown.

In this study, we investigated the role of ZBED6 in cardiac hypertrophy and its underlying mechanism. We found that ZBED6 expression is markedly reduced in myocardial tissues from patients with cardiac hypertrophy and in hypertrophic cardiomyocytes. Using ZBED6-deficient pigs and human cardiomyocytes, we demonstrated that loss of ZBED6 causes cardiac hypertrophy through autophagy impairment. Mechanistically, we identified MEOX1 as a negative transcriptional target of ZBED6 in the heart. ZBED6 depletion increases MEOX1 transcription, which activates the MAPK/ERK1/2 pathway, inhibits autophagy, thereby drives hypertrophy. Conversely, *MEOX1* knockdown or ERK1/2 inhibition restored autophagy and alleviated ZBED6 deficiency–induced hypertrophy. Together, our findings define a previously unrecognized ZBED6-MEOX1-MAPK/ERK1/2 regulatory axis in cardiomyocyte hypertrophy and establish ZBED6 as a potential therapeutic target.

## Results

### ZBED6 is downregulated in humans with cardiac hypertrophy and hypertrophic cardiomyocytes

ZBED6 protein and mRNA levels were significantly reduced in myocardial tissue from patients with cardiac hypertrophy (Fig 1A and 1B). To model hypertrophic stress *in vitro*, AC16 cells were treated with Angiotensin II (Ang II), which induced a robust increase in cell size (Fig 1C and 1D) and a marked decrease in ZBED6 protein levels (Fig 1E). These results demonstrate that ZBED6 is consistently downregulated in cardiac hypertrophy in humans and human cardiomyocytes, implicating ZBED6 loss as a potential driver of pathological cardiac hypertrophy.

**Fig 1:**
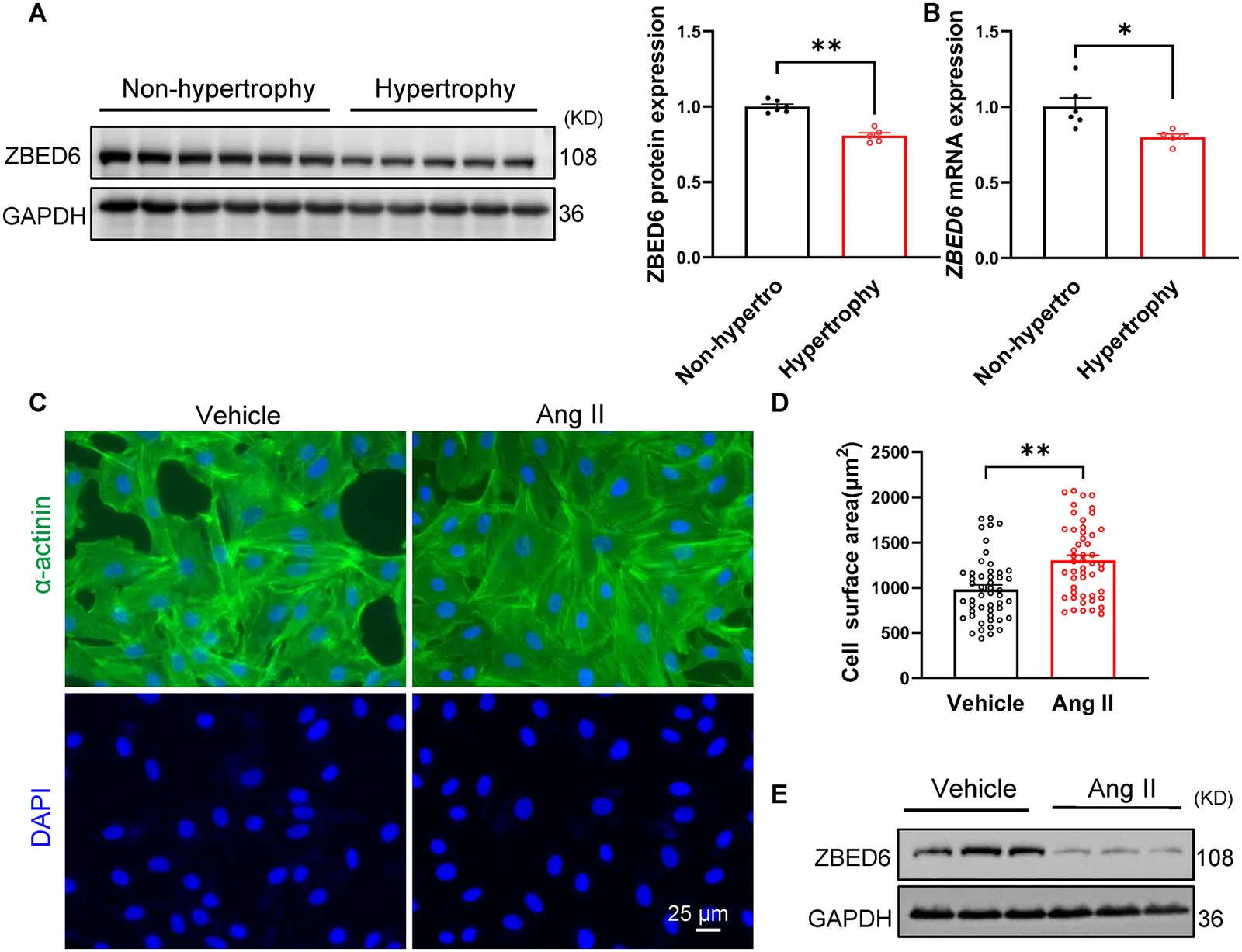
ZBED6 expression is downregulated in cardiac hypertrophy patients and cardiomyocytes subjected to hypertrophic stress. **(A)** Immunoblotting and quantitation of ZBED6 protein level of cardiac tissue in healthy individuals (n = 6) and cardiac hypertrophy patients (n = 5). **(B)** The mRNA level of *ZBED6* were analyzed by RT-qPCR and normalized to *GAPDH.* **(C and D)** Representative immunofluorescence images of α-actinin and quantification of AC16 cell size treated with Ang II (1 μM) or vehicle for 24 hours (n = 50). Scale bars: 25 μm. **(E)** ZBED6 protein level in AC16 cells treated with vehicle or Ang II for 24h (n = 50). Data are presented as mean ± SEM; * *P* < 0.05, ** *P* < 0.01.

### ZBED6 deficiency induces cardiac hypertrophy with diastolic dysfunction in pigs

To define the functional role of ZBED6 in cardiac hypertrophy *in vivo*, we generated ZBED6-deficient pigs (*ZBED6*^-/-^) as previously described [10] and confirmed complete absence of ZBED6 protein in the heart (S1A Fig). Echocardiography at 9 months of age revealed preserved systolic function in *ZBED6*^-/-^ pigs, as evidenced by stable left ventricular ejection fraction (LVEF) and fractional shortening (LVFS) (Fig 2A-2C). In contrast, pulse-wave Doppler and tissue Doppler echocardiography analyses demonstrated impaired diastolic function, reflected by a significantly decreased E/A ratio (Fig 2D and 2E) and a markedly increased E/e′ ratio (Fig 2F and 2G). To determine whether the impaired diastolic function observed in vivo was accompanied by intrinsic alterations in cardiomyocyte contractile properties, we next assessed contraction and relaxation dynamics in isolated primary porcine cardiomyocytes. ZBED6 deficiency had no significant effect on contraction time or contraction velocity (S1B–S1D Fig). In contrast, *ZBED6*^-/-^ cardiomyocytes exhibited prolonged relaxation time and reduced relaxation velocity compared with WT cardiomyocytes (S1B, S1C and S1E Fig). Fractional shortening and resting sarcomere length were comparable between WT and *ZBED6*^-/-^ cardiomyocytes (S1F–S1G Fig). The contractile and relaxation characteristics of ZBED6-deficient primary cardiomyocytes were similar to those observed in cardiomyocytes from HFpEF hearts. Together, these findings indicate that ZBED6 deficiency induces cardiac hypertrophy accompanied with impaired diastolic function.

**Fig 2:**
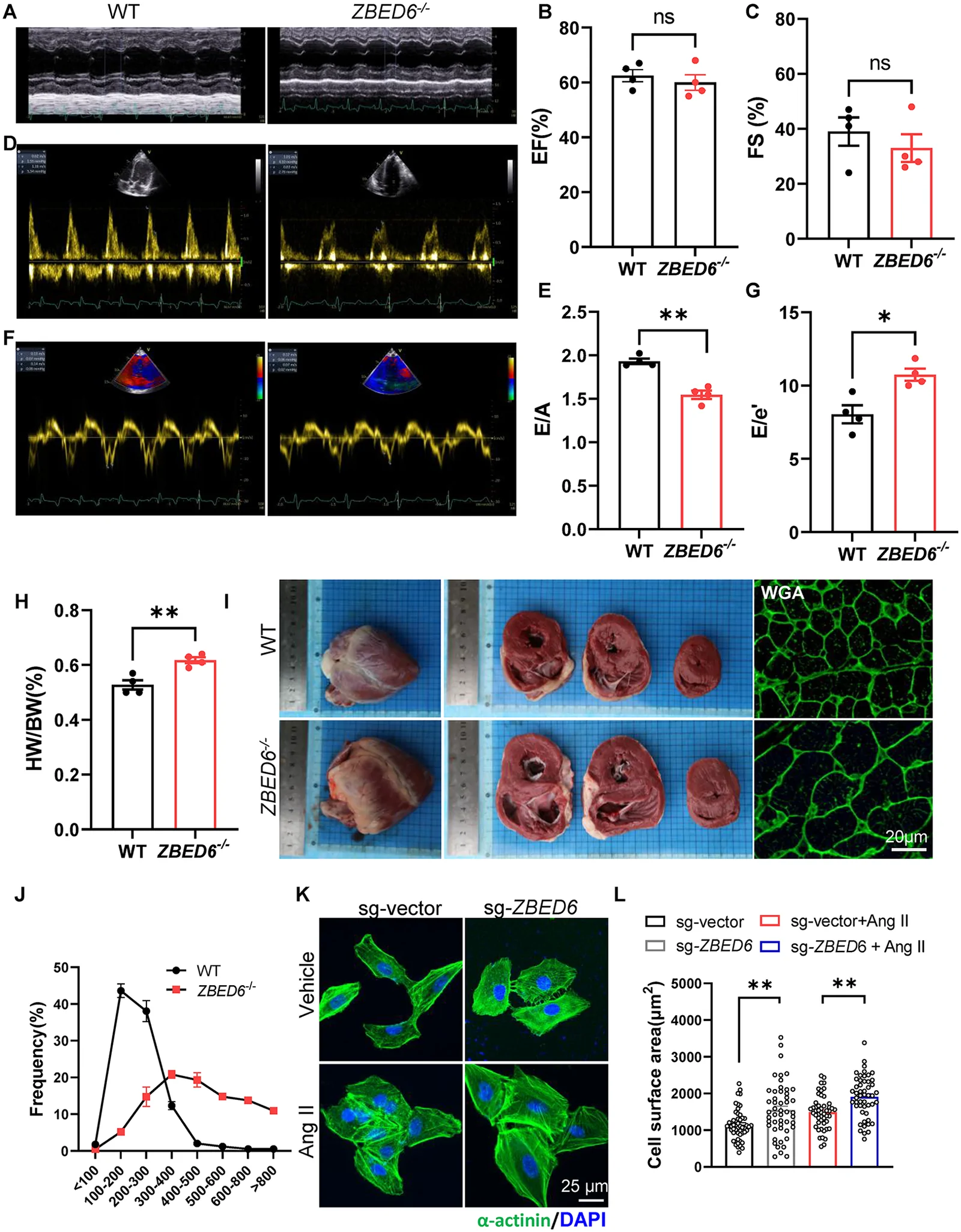
ZBED6 deficiency drives cardiac hypertrophy and diastolic dysfunction in pigs. **(A)** Representative images of longitudinal echocardiography (Echo) from 9-month-old *ZBED6*-deficient and WT pigs. **(B and C)** Echo parameters of panel A were calculated (n = 4). LVEF: left ventricular ejection fraction in **(B)**, LVFS: left ventricular fraction shortening in **(C)**. **(D and E)** Representative images of pulse-wave Doppler in (D) and the ratio between mitral E wave and A wave (E/A) **(E)**. **(F and G)** Representative tissue Doppler tracings in (F) and quantitative analysis of the mitral E to e′ -wave ratio (E/e′) in **(G)**. **(H)** The ratio of wet heart weight to body weight (HW/BW) (n = 3). **(I)** Representative photographs of general hearts, transverse cross sections and WGA-stained sections of heart in each group at 9 months (n = 3). Scale bars, 50 μm. **(J)** Frequency distribution of myofiber cross-sectional area (μm^2^) in the left ventricle for each group (n = 3). **(K and L)** Representative α-actinin immunofluorescence images and quantification of relative cross-sectional area in WT and *ZBED6*-deficient cells treated with vehicle or Ang II (1 μM) for 24 h (n = 50). Scale bars, 25 μm. Data are expressed as mean ± SEM; * *P* < 0.05, ** *P* < 0.01, ns, not significant.

Morphological analyses further demonstrated that ZBED6 deficiency caused pronounced cardiac remodeling. *ZBED6*^-/-^ pigs displayed significantly increased heart weight and volume (Fig 2H–2I), left ventricular chamber narrowing, and myocardial wall thickening (Figs 2I, S1H–S1M). Wheat germ agglutinin staining revealed significant cardiomyocyte enlargement in *ZBED6*^-/-^ hearts (Fig 2I–2J), suggesting that increased cell size is the primary contributor to organ-level hypertrophy.

To test whether ZBED6 directly regulates cardiomyocyte size, CRISPR/Cas9-mediated deletion of ZBED6 in human cardiomyocytes resulted in a significant increase in cell size, which was further amplified under Ang II stimulation (Figs 2K-2L, S2A and S2B). Correspondingly, the mRNA levels of the hypertrophic markers *ANP*, *BNP* and *MYH7*, as well as the protein level of ANP, were markedly elevated in ZBED6-deficient cells treated with either PBS or Ang II. (S2C–S2E Fig). Similarly, mRNA levels of *ANP*, *BNP* and protein levels of ANP were significantly upregulated in ZBED6^-/-^ pig hearts (S3A and S3B Fig). The longitudinal section analysis of myocardial fibers revealed slightly myofiber disarray in ZBED6-KO hearts compared with wild-type controls (S3C Fig). Masson’s trichrome staining revealed mild interstitial fibrosis in *ZBED6*^-/-^ pig hearts (S3D–S3F Fig). Collectively, these findings demonstrate that ZBED6 deficiency causes pathological cardiac hypertrophy, impaired diastolic function, and molecular remodeling in pigs.

### Loss of *ZBED6* induces cardiac hypertrophy through autophagy impairment

To test whether ultrastructural remodeling of cardiac tissue contributes to ZBED6 deficiency-induced cardiac hypertrophy, mitochondria morphology assessed by transmission electron microscopy (TEM) and function as shown by oxygen consumption rate (OCR), as well as respiratory chain protein and mRNA levels were evaluated, the results were comparable between ZBED6- deficient and WT cardiomyocytes (S4A–S4E Fig). However, we noticed a marked accumulation of autophagic vesicles in ZBED6-deficient porcine cardiac tissues, identified by double-membrane structure enclosing cytoplasmic content or organelles (Fig 3A), indicative of impaired autophagic flux. This was accompanied by elevated protein levels of autophagosome marker LC3B and p62, a key mediator and substrate of selective autophagy, in ZBED6-deficient hearts (Fig 3B). Similar findings were shown in ZBED6-knockout AC16 cardiomyocytes (Fig 3C). Consistent with these findings, colocalization of LC3B puncta with LAMP1-positive lysosomes was significantly reduced in ZBED6-deficient human cardiomyocyte (Fig 3D). Collectively, these observations suggested that the accumulation of autophagic vesicles may reflect impaired autophagic clearance.

**Fig 3:**
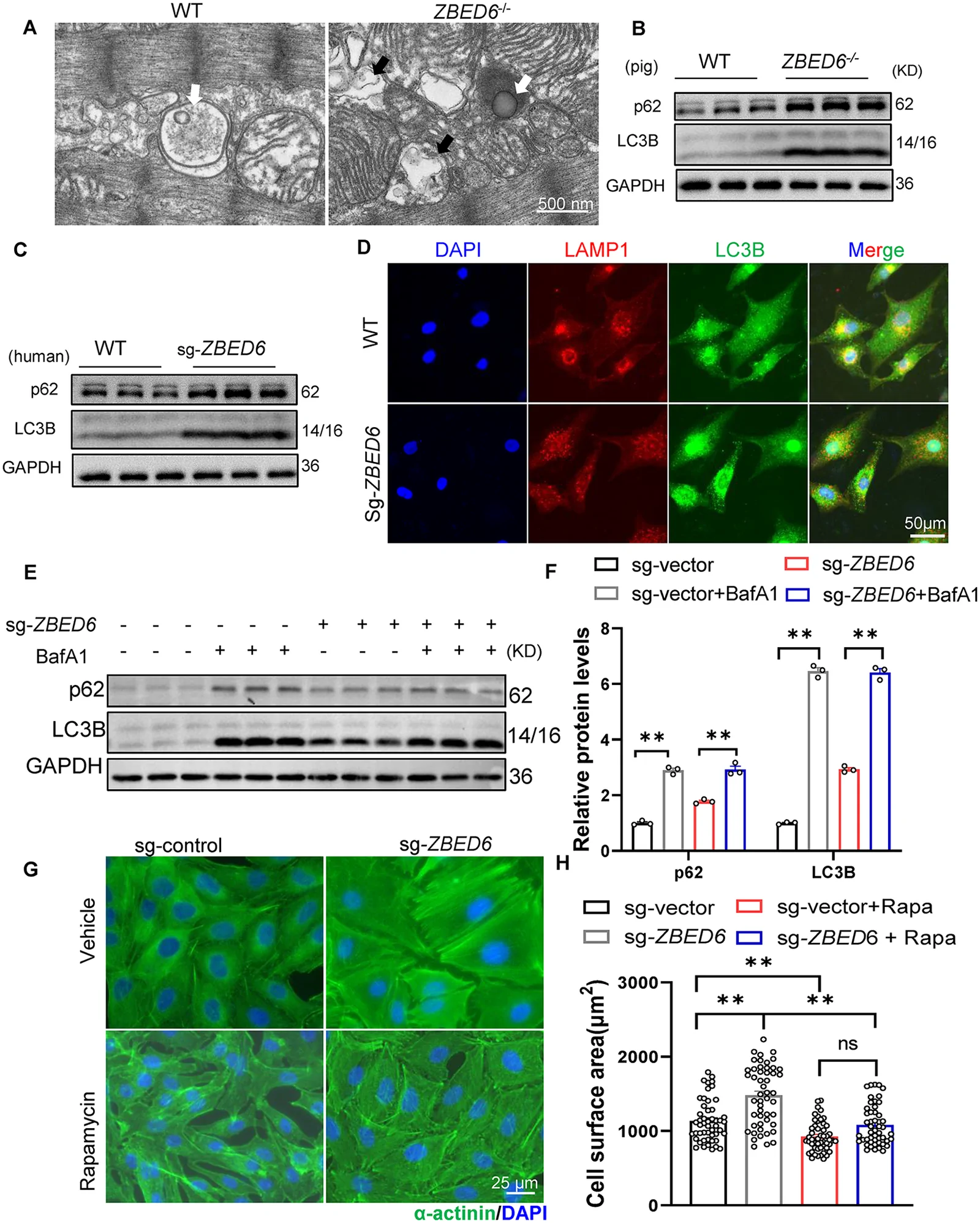
Loss of ZBED6 induces cardiac hypertrophy through autophagy impairment. **(A)** Representative electron microscopic images of autophagy in left ventricular myocardial from *ZBED6*-deficient and wild-type pigs. (White arrow indicated autolysosomes, black arrow autophagosome). Scale bars, 500 nm. **(B and C)** The protein levels of P62 and LC3B in *ZBED6*-deficient pigs (B) and *ZBED6*-depletion cardiomyocytes (C) compared with control (n = 3). **(D)** Representative immunofluorescence images of wild-type and *ZBED6*-deficient cardiomyocytes stained for LC3B (green), LysoTracker (red), and nuclei (blue). Scale bars, 50 μm. **(E and F)** Relative protein expression and quantification of LC3B and p62 in WT and ZBED6-dificient AC16 cells treated with BafA1 (50 nM) or DMSO for 24 hours (n = 3). **(G and H)** Representative immunofluorescence images of α-actinin staining and quantification of cell surface area in WT and *ZBED6*-deficient AC16 cells treatment with DMSO or Rapamycin (2 μM) for 24 hours (n = 50). Scale bars, 25 μm. Data are presented as mean ± SEM. *P < 0.05, **P < 0.01.

To further determine whether the accumulation of autophagic vesicles in ZBED6-deficient cardiomyocytes reflected enhanced autophagosome formation or impaired autophagic clearance, we assessed autophagic flux using Bafilomycin A1 (BafA1), which inhibits lysosomal degradation. Under basal conditions, ZBED6-deficient cells exhibited increased LC3B and p62 protein levels compared with control cells (Fig 3E and 3F). BafA1 treatment markedly increased LC3B and p62 levels in both control and ZBED6-deficient cells. However, the BafA1-induced accumulation of these autophagic markers was less pronounced in ZBED6-deficient cells than in control cells, particularly for LC3B (~2.2 fold versus ~6.4 fold), suggesting reduced autophagic turnover following ZBED6 loss (Fig 3E and 3F). Together, these findings indicate that ZBED6 deficiency impairs autophagic flux rather than simply enhancing autophagosome formation. Pharmacological activation of autophagy with rapamycin attenuated ZBED6 deficiency-induced hypertrophic phenotype (Fig 3G and 3H). Autophagy inhibition is accompanied by apoptosis increase in pathological cardiac hypertrophy [25]. To further investigate whether ZBED6 deficiency affects apoptosis, we performed TUNEL staining in cardiac tissues and cardiomyocytes. We found a significant increase in apoptosis in ZBED6-deficient hearts and cardiomyocytes (S5A–S5D Fig). Collectively, these data indicate that ZBED6 deficiency promotes cardiac hypertrophy by impairing autophagy and apoptosis.

### Integrative RNA-seq and ChIP-seq analysis identified MEOX1 as a direct cardiac target of ZBED6

To uncover downstream mediator of ZBED6 in cardiac hypertrophy, we performed an integrative analysis of RNA-seq data from *ZBED6*^-/-^ and WT pig hearts with ZBED6 ChIP-seq profiles, yielding 133 potential ZBED6 targets. To enhance the translational relevance, these candidate genes were further cross-referenced with three human cardiac hypertrophy GEO datasets, resulting in 18 conserved, differentially expressed genes with promoter-proximal ZBED6 binding (Fig 4A). Six (*MEOX1*, *BDNF*, *MCF2L2*, *ARHGAP28, AGRE, CX3CR1*) of the 18 genes exhibited consistent expression patterns in both human and porcine hypertrophic myocardium (S6A Fig). qPCR validation confirmed that *BDNF*, *MEOX1*, *ARHGAP28* and *MCF2L2* were significantly upregulated in *ZBED6*^-/-^ pig hearts, whereas *AGRE* and *CX3CR1* were unchanged (Fig 4B).

**Fig 4:**
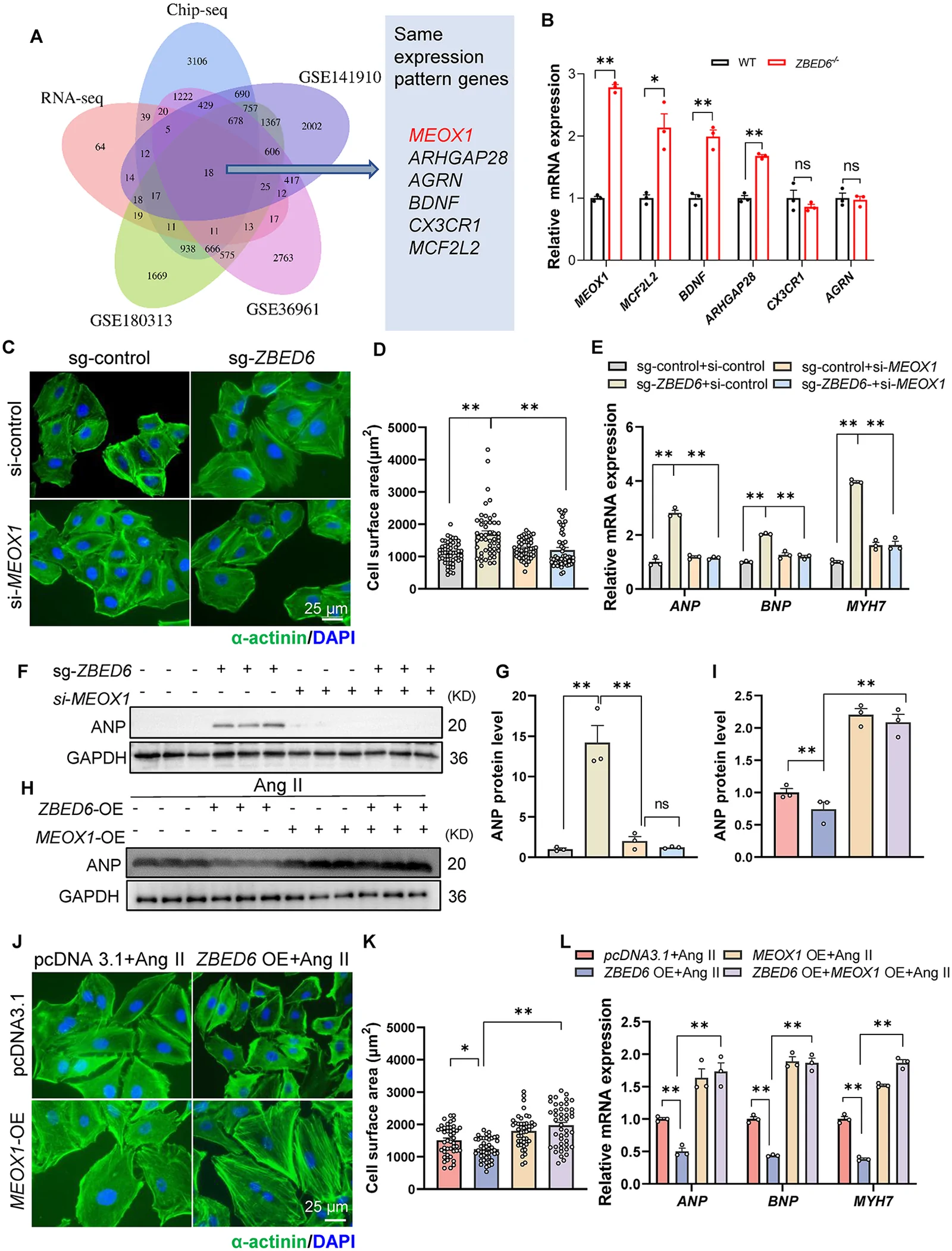
ZBED6-MEOX1 axis regulates cardiomyocyte hypertrophy. **(A)** Integrated analysis of cardiac RNA-seq and ChIP-seq data from WT and ZBED6-deficient pigs, combined with human RNA-seq datasets, identifies six potential ZBED6 targets. **(B)** Relative mRNA levels of *MEOX1*, *MCF2L2*, *BDNF*, *ARHGAP28*, *CX3CR1* and *AGRN* in WT and *ZBED6* knockout pigs (n = 3). **(C and D)** Representative immunofluorescence images α-actinin and quantification of cell surface area in WT and *ZBED6*-deficient AC16 cells transfected with siNC or si*MEOX1*, stained for α-actinin (green) and nuclei (blue) (n = 50). Scale bar: 25 μm. **(E)** The relative mRNA levels of *ANP*, *BNP* and *MYH7* in WT and ZBED6-deficient AC16 cells under the indicated groups (n = 3). **(F and G)** Immunoblot analysis and quantification of ANP protein level from samples (n = 3). (**H** and **I**) Western blot analysis and quantification of ANP in each group (n = 3). **(J and K)** Representative immunofluorescence images α-actinin and quantification of cell surface area in WT and *ZBED6*-OE cells transfected with pcDNA3.1 or *MEOX1*-overexpression vector, followed by Ang II treatment (n = 45). Scale bar: 25 μm. **(L)** The mRNA levels of *ANP*, *BNP* and *MYH7* in WT and *ZBED6*-overexpression AC16 cells under the indicated groups (n = 3). Data are expressed as mean ± SEM; * *P* < 0.05, ** *P* < 0.01, ns, not significant.

Dual-luciferase assays demonstrated that ZBED6 suppresses transcriptional activity of the *MEOX1*, *BDNF*, and *MCF2L2*, but not *ARHGAP28* (S6B Fig). Among the three candidates, only *MEOX1* knockdown mitigated ZBED6 deficiency-induced hypertrophy, reducing cardiomyocyte size (Figs 4C and 4D, S6C–S6E), suppressing the expression of hypertrophic markers *ANP*, *BNP* and *MYH7* mRNA levels as well as ANP protein levels (Fig 4E–4G). Conversely, *MEOX1* overexpression abrogated the anti-hypertrophic effect of *ZBED6* overexpression (Fig 4H–4L). These results suggest that ZBED6 may protect against cardiac hypertrophy by inhibiting the expression of MEOX1. Consistent with these findings, the levels of *MEOX1* mRNA and protein were significantly upregulated in *ZBED6*-depleted human cardiomyocytes (S7A and S7B Fig) and *ZBED6*^-/-^ pig myocardial tissue (S7C Fig). Hypertrophic human hearts also displayed elevated MEOX1 accompanied with reduced ZBED6 (Figs 1A, S7D). These results suggest that MEOX1 is a potential direct target of ZBED6 and that loss of ZBED6 drives cardiac hypertrophy through upregulating MEOX1 expression.

We further identified a conserved “GCTCG” ZBED6-binding motif (ZBS) in the promoter region of porcine *MEOX1* (S7E Fig). We constructed a MEOX1-luciferase reporter containing the *MEOX1* promoter region from 0 to -2000 bp and performed transfection experiments in 293T cells. *ZBED6* overexpression decreased MEOX1 promoter activity (~64% reduction) (S6B Fig), whereas mutation of this motif significantly increased the luciferase activity by approximately 4-fold (S7F Fig). ChIP-PCR confirmed that ZBED6 directly binds to the endogenous *MEOX1* promoter in pig heart (S7G Fig), and luciferase activity was enhanced (∼3-fold) in ZBED6-deficient AC16 cells (S7H Fig). Together, these findings identify *MEOX1* as a direct, transcriptionally repressed target of ZBED6 and a key mediator of ZBED6-dependent cardiomyocyte hypertrophy.

Previous studies have identified IGF2 as a direct and functionally important target of ZBED6 in myogenesis. To determine whether IGF2 contributes to ZBED6 deficiency-induced cardiomyocyte hypertrophy, we knocked down IGF2 in ZBED6-deficient cardiomyocytes (S8A–S8C Fig). However, *IGF2* knockdown did not attenuate the cardiomyocyte hypertrophy induced by ZBED6 deficiency (S8D and S8E Fig), suggesting that this hypertrophic phenotype is largely independent of *IGF2*.

### ZBED6-MEOX1 axis activates MAPK-ERK1/2 signaling, disrupts autophagy and thereby induces cardiac hypertrophy

To further elucidate the downstream pathway of ZBED6, we performed Gene Set Enrichment Analysis (GSEA) and identified MAPK signaling as significantly enriched pathway in *ZBED6*^-/-^ pig hearts (Fig 5A). The MAPK signaling pathway has been shown to be closely associated with autophagy dysfunction [26] and cardiac hypertrophy [27]. Among the three principal MAPK pathways–ERK1/2, p38, and JNK, only ERK1/2 was selectively activated in the cardiac tissue from ZBED6-deficient pigs (Fig 5B). ERK1/2 activation was also shown in patients with cardiac hypertrophy (Fig 5C). Given the established role of ERK1/2 in autophagy dysfunction and cardiac hypertrophy, these findings suggest that ZBED6 deficiency may contribute to cardiac hypertrophy through activation of ERK1/2 pathway.

**Fig 5:**
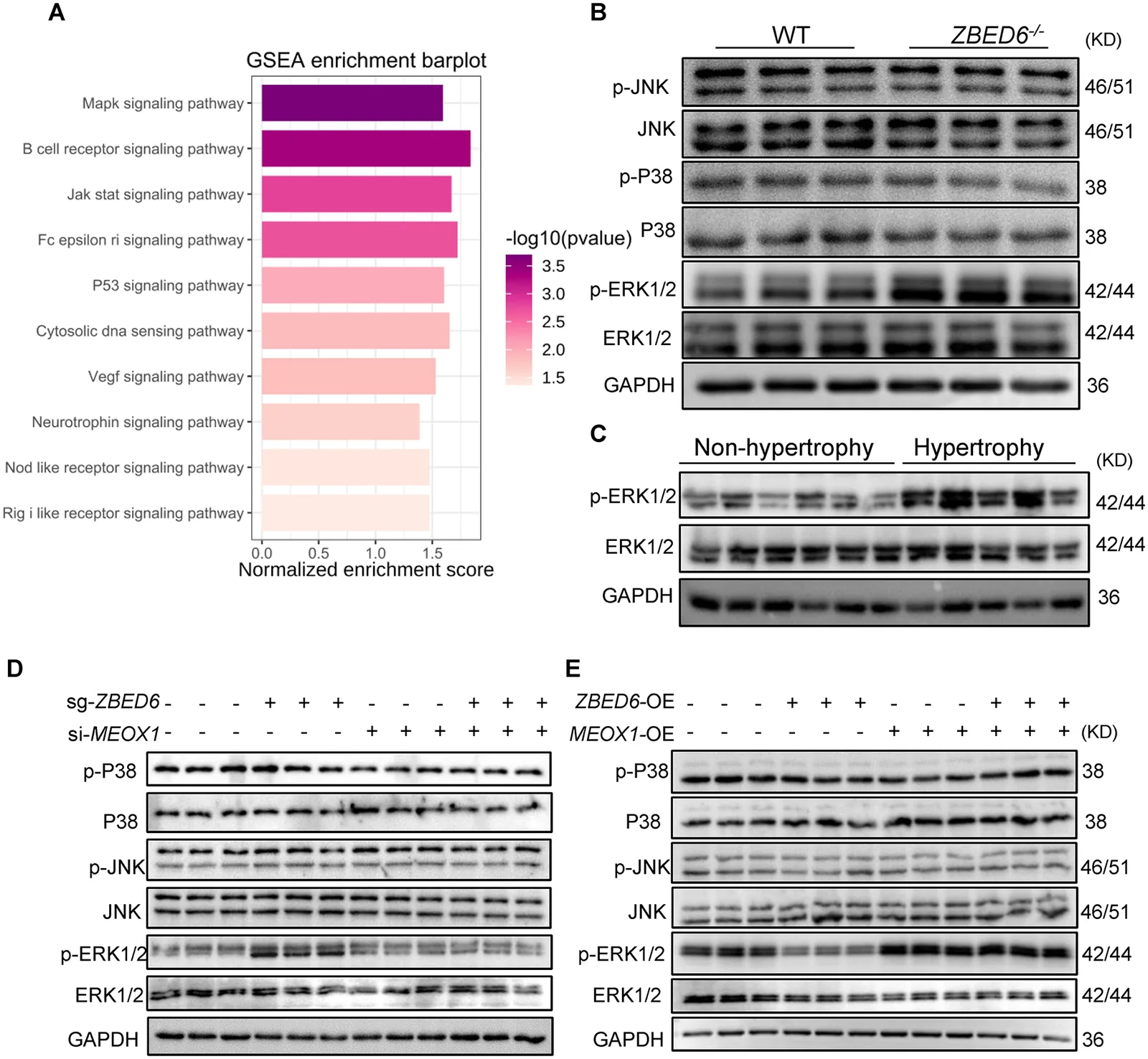
ZBED6 regulates cardiac hypertrophy through the MEOX1-MAPK signaling pathway. **(A)** GSEA identified enriched signaling pathways in *ZBED6*-deficient versus WT pig cardiac transcriptomes. **(B)** The protein levels of key MAPK pathway kinases in cardiac tissue of WT and ZBED6-dificient pigs (n = 3). **(C)**. The protein levels of phosphorylated ERK1/2 (p-ERK1/2) and total ERK1/2 in cardiac tissue from healthy individuals (n = 6) and cardiac hypertrophy patients (n = 5). **(D)** Western blot analysis of key MAPK pathway kinases in WT or ZBED6 knockout (KO) AC16 cells transfected with siNC or si*MEOX1* (n = 3). **(E)** Western blot analysis of key MAPK pathway kinases in WT and *ZBED6*-OE cells transfected with pcDNA3.1 or *MEOX1*-overexpression vector, followed by Ang II treatment (n = 3).

To further determine whether activation of ERK1/2 by ZBED6 deletion is MEOX1 dependent, we constructed ZBED6-deficient cardiomyocytes and found significantly elevated protein levels of p-ERK1/2 and MEOX1, without detectable effects on the p38 or JNK pathways (Figs 5D, S7B). Knockdown of *MEOX1* suppresses ERK1/2 activation in ZBED6-deficient cardiomyocytes (Fig 5D), whereas *ZBED6* overexpression reduced p-ERK1/2 protein levels (Fig 5E). Overexpression of *MEOX1* in *ZBED6*-overexpressing cells attenuated the inhibitory effect of ZBED6 on ERK1/2 phosphorylation (Fig 5E). Consistently, left ventricular tissues from patients with cardiac hypertrophy exhibited decreased ZBED6 levels, increased MEOX1 expression and p-ERK1/2/T-ERK1/2 ratios (Figs 1A and 5C, S7D). Collectively, these findings demonstrate that ZBED6 deficiency promotes cardiac hypertrophy by unleashing MEOX1-dependent ERK1/2 activation.

### ERK1/2 Inhibition rescues ZBED6 deficiency-induced autophagy defects and cardiomyocyte hypertrophy

The ERK1/2 signaling pathway is one of the keys signaling cascades in cardiac hypertrophy [28, 29]. Here *ZBED6* deletion activated the MEOX1-ERK1/2 axis, leading to cardiac hypertrophy. This prompts us to investigate whether inhibiting ERK1/2 phosphorylation could alleviate the autophagy dysfunction-induced hypertrophy in ZBED6-deficient cardiomyocytes. Pharmacological inhibition of ERK1/2 with SCH772984 (1 μM) effectively reversed the enlarged cell size of ZBED6-deficient cardiomyocytes (Fig 6A-6B) and significantly reduced *ANP, BNP*, and *MYH7* mRNA levels, as well as ANP protein levels caused by ZBED6 deficiency (Fig 6C-6E). SCH772984 attenuated the LC3B and p62 levels in ZBED6-deficient cardiomyocytes (Fig 6E), indicating restoration of autophagic flux. We further found that the protective effect of ERK1/2 inhibition was abolished by BafA1, an autophagy inhibitor (Fig 6F-6G)**,** confirming that the reversal of hypertrophy requires restoration of autophagy. Together, these results establish that the ZBED6–MEOX1–ERK1/2 axis promotes cardiac hypertrophy by disrupting autophagy, and that ERK1/2 blockade rescues these defects.

**Fig 6:**
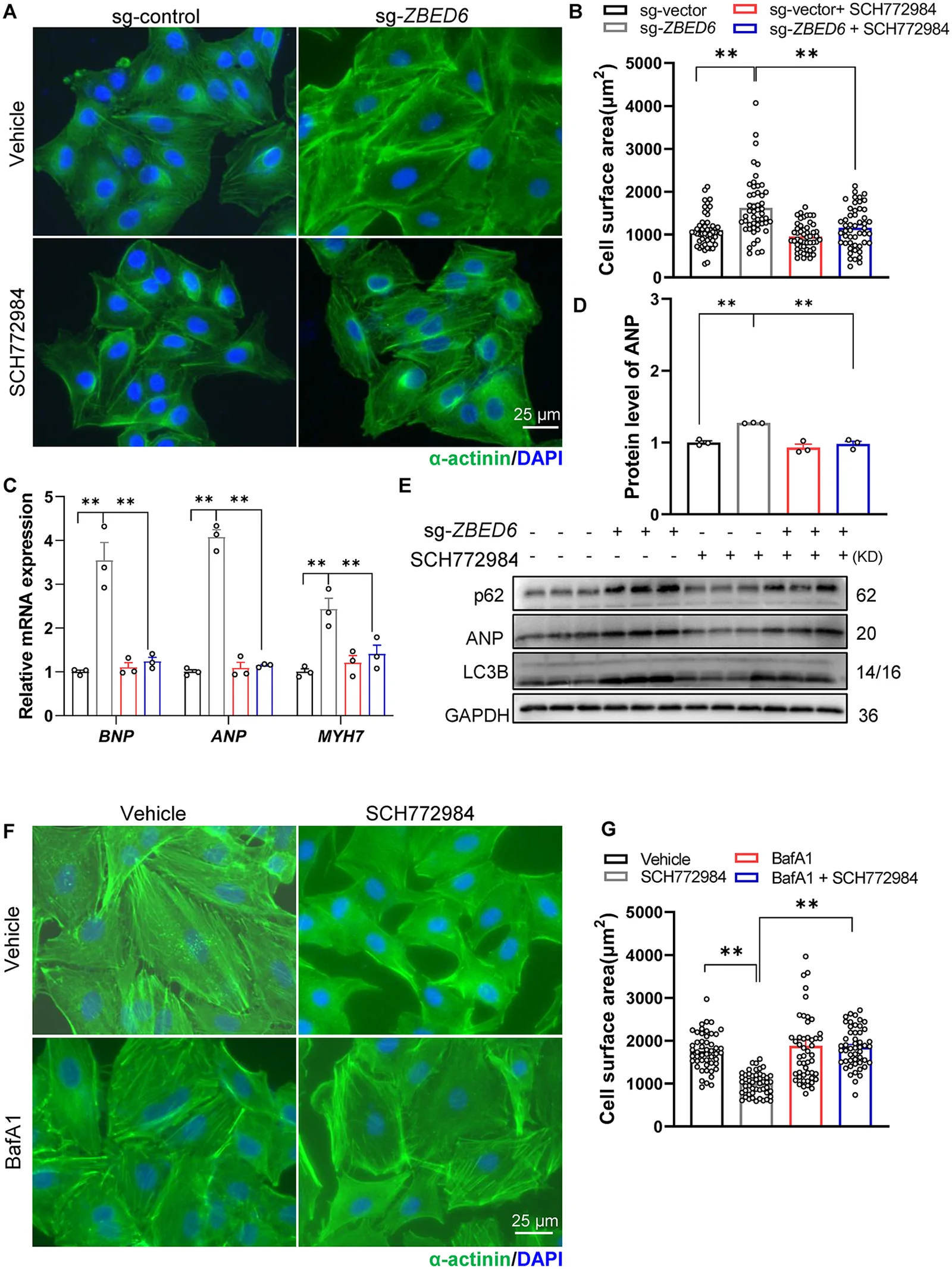
ZBED6 deficiency-induced cardiomyocyte hypertrophy is reversed by inhibition of MEOX1-induced pERK1/2 activation. **(A)** Representative immunofluorescence images of α-actinin in WT and ZBED6-deficient AC16 cells treated with vehicle or SCH772984 for 48 hours. Scale bar, 25 μm. **(B)** Quantification of cardiomyocyte cross-sectional area in A (n = 50). **(C)** Relative mRNA levels of *ANP*, *BNP* and *MYH7* in WT and *ZBED6*-deficient AC16 cells treated with vehicle or SCH772984 (n = 3). **(D and E)** Immunoblot analysis of ANP, LC3B and p62 in wild type and *ZBED6*-deficient AC16 cells subsequently treated with vehicle or SCH772984 (n = 3). **(F and G)** Representative α-actinin immunofluorescence images and quantification of the cell size in *ZBED6*-deficient AC16 cells treated with either SCH772984 or BafA1 for 48 hours (n = 50). Scale bar, 25 μm. Data are expressed as mean ± SEM; * *P* < 0.05, ** *P* < 0.01, ns, not significant.

## Discussion

Pathological cardiac hypertrophy is a major contributor to heart failure and associated sudden death [1]. Here, we identify the transcription factor ZBED6 as a previously unrecognized regulator of cardiac hypertrophy. Genetic deletion of ZBED6 in pigs—a large-animal model physiologically relevant to humans—induces left ventricular hypertrophy, diastolic dysfunction, cardiomyocyte enlargement, and mild fibrosis, firmly establishing ZBED6 as a protective factor against pathological hypertrophy. Mechanistically, integrated transcriptomic and ChIP-seq analyses identified MEOX1 as a direct transcriptional target of ZBED6, and downstream activation of ERK1/2 as the key signaling pathway linking ZBED6 deficiency to impaired autophagy and hypertrophy. Both genetic and pharmacological interference with the ZBED6–MEOX1–ERK1/2 pathway attenuated hypertrophy by restoring autophagy, revealing a previously unexplored mechanism underlying cardiac pathology, and provide new therapeutic options for treating pathological cardiac hypertrophy.

Although increased heart weight has been previously observed in ZBED6-defient pigs and mice, this study provides the first demonstration that ZBED6 loss results in pathological, rather than physiological, hypertrophy. This hypertrophic phenotype aligns with that driven by defects in the classical sarcomeric genes such as *MYH7*, *TNNT2* and *MYBPC3*, whereas ZBED6 regulates hypertrophy indirectly through transcriptional control of hypertrophy gene expression. Thus, our findings broaden the current understanding of the cardiac regulatory network by identifying ZBED6 as a transcriptional “brake” whose loss promotes pathological cardiac hypertrophy. The large-animal model established here provides a translationally relevant platform for investigating human hypertrophic cardiomyopathy and diastolic dysfunction.

ZBED6 is known to regulate distinct gene sets depending on tissue context and physiological or pathological state [9]. While IGF2 is its canonical target in regulating the growth of skeletal muscle under physiological conditions [10,11]. Our previous work on ZBED6 protects against sepsis-induced muscle atrophy, revealed DOCK3 as its target during this process [12]. In this study, our integrative analysis identifies MEOX1 as its direct target in the heart during pathological remodeling. Thus, it is conclusive that the regulatory role of ZBED6 has distinct tissue/organ specificity under different conditions. Although MEOX1 has been implicated as a driver of cardiac hypertrophy [13,14], its upstream transcriptional regulation was previously unknown. Our work fills this gap by identifying ZBED6 as a negative regulator of MEOX1 and demonstrating that MEOX1 derepression is necessary and sufficient to trigger ERK1/2 activation and hypertrophy in the context of ZBED6 deficiency.

Downstream of MEOX1, we established ERK1/2 as the pivotal effector linking ZBED6 loss to autophagy impairment and hypertrophy. Consistent with prior work that pharmacologic ERK1/2 inhibition suppresses hypertrophy [21], we demonstrate that SCH772984 treatment rescues both autophagic flux and cardiomyocyte enlargement in ZBED6-deficient cells. These findings highlight ERK1/2 not merely as a downstream effector of hypertrophic stress, but aslo as a key mediator of autophagy disruption and cardiac hypertrophy within the ZBED6–MEOX1 regulatory axis.

Several cellular events have been implicated in the development and progression of cardiac hypertrophy, such as calcium-handling dysfunction [30], autophagy [18,31] or mitochondrial dysfunction [32,33]. While mitochondrial function appeared largely preserved in ZBED6-deficient hearts, autophagic flux was markedly impaired, as evidenced by autophagosome accumulation, elevated LC3B and p62 levels, and defective autophagosome–lysosome fusion. ERK1/2 inhibition restored autophagy and reversed hypertrophy, establishing autophagy dysfunction as a mechanistic driver of pathological growth in this model.

Several limitations warrant consideration. Although pigs provide a powerful translational model, further validation of the ZBED6–MEOX1–ERK1/2 axis in primates or human cardiomyocyte systems derived from patient samples will strengthen its clinical relevance. In addition, while our study primarily focuses on molecular mechanisms of cardiac hypertrophy, systemic consequences—such as renal or circulatory complications—remain to be explored.

In summary, this work reveals a previously unrecognized role of ZBED6 as a transcriptional suppressor of cardiac hypertrophy and identifies a mechanistic pathway in which ZBED6 loss derepresses MEOX1, activates ERK1/2, disrupts autophagy, and drives pathological remodeling. These findings uncover ZBED6 as a promising therapeutic target and expand the current framework for understanding the molecular pathogenesis of hypertrophic cardiomyopathy.

## Materials and methods

### Ethics Statement

All specimens were processed immediately after surgery and stored at less than −80 °C until protein were extracted. The Ethics Institutional Review Board of Shanghai Jiao Tong University approved all human heart tissue samples conducted in this research, with clinical ethical approval (SH9H-2018-T45-3). Animal studies were approved by the Institutional Animal Care and Use Committee of Northwest A and F University accredited by AAALAC International. All applicable institutional and national guidelines for the care and use of animals were followed.

**Animals.** Breeding and rearing of Bama miniature pigs were performed as previously described [12]. For echocardiographic studies, the operators were blinded from the animal genotypes, and animal numbers were estimated by the pilot experiments or published protocols.

**Human heart samples.** The myocardium samples used in this study were obtained from 5 patients previously diagnosed with HCM and 6 healthy donors, undergoing septal myectomy surgery. Informed written consent to participate in research was obtained from patients and donors.

**Wheat germ agglutinin (WGA) staining and cardiomyocyte cross-sectional area quantification.** Left ventricular tissues were fixed in 4% paraformaldehyde, embedded in paraffin, and transversely sectioned at a thickness of 5 μm. After deparaffinization and rehydration, sections were incubated with fluorescently labeled wheat germ agglutinin (WGA) at 5 μg/mL for 30 min at room temperature and counterstained with DAPI. Images were acquired from randomly selected, non-overlapping fields of the left ventricular myocardium. Only transversely sectioned cardiomyocytes with intact continuous WGA borders were included. Obliquely sectioned, torn, overlapping, or incompletely bordered cells were excluded. Cardiomyocyte boundaries were manually delineated along the WGA-stained membrane in ImageJ, and the enclosed area of each individual cardiomyocyte was automatically calculated by the software. Quantification was performed by investigators blinded to genotype. At least five fields per section and at least 200 cardiomyocytes per animal were analyzed.

**Pig cardiomyocyte isolation.** Pig cardiomyocytes were isolated from hearts in 9-month-old WT and ZBED6-deficient pigs as previously described [34]. Once anesthesia, the heart was removed and perfused with Ca^2+^-free Tyrode’s solution containing 300 U/ml collagenase type II at 37 °C for 1 h by using Langendorff perfusion system. After perfusion, the portions of the left ventricular free wall were collected and triturated. Cardiomyocytes were dispersed by using pipette, filtered with a 200 μm-nylon mesh, and collected by centrifuged (100 × g, 5 min, 4˚C). Collected cardiomyocytes were resuspended in Ca^2+^-free Tyrode’s solution with 10% FBS to further analyses.

**Pig cardiomyocyte contractility.** Isolated pig cardiomyocyte contractility was assessed [35]. Cardiomyocytes were transferred in a fabricated cell chamber (IonOptix) mounted on an Zeiss 880 inverted confocal microscope with either a 60 x oil objective and transmitted light camera (IonOptix) and were paced using field stimulation at 1Hz. The contraction traces were recorded and analyzed.

**TUNEL staining assay.** Apoptosis in cultured cardiomyocytes and cardiac tissues was detected using a One Step TUNEL Apoptosis Assay Kit with red fluorescence (Beyotime Biotechnology, C1089) according to the manufacturer’s instructions. Cultured cardiomyocytes were subjected to TUNEL staining and counterstained with DAPI. Cardiac tissue sections were stained with TUNEL, an anti-cardiac troponin T antibody (Proteintech, 15513-1-AP), and DAPI. Images were obtained from randomly selected, non-overlapping fields and analyzed using ImageJ/Fiji. The percentage of apoptotic cells was calculated as the number of TUNEL-positive nuclei divided by the total number of DAPI-positive nuclei.

**Generation of ZBED6 KO cell lines.** AC16 Cells transfected with 2 ZBED6-sgRNAs expression vector after 3 days were selected for puromycin-resistant clones by incubating them in media containing puromycin for a period of 3 days at 37 °C / 5% CO_2_, with media changes every day until the colonies were large enough to be picked. Single cell clones were processed into small clumps using a 20–200μl pipette tip and cultured into 24 well plates. At least 24 colonies were picked for genotyping to confirm the knockouts clones by sequencing PCR products of the edited regions. PCR primers were as follows: forward (5′- CCTGGCTTGGAAGTTATTG-3′) and reverse (5′-GCAGATGTCGTTGAAGAGTAG-3′). The deletion of ZBED6 was validated by Western blot assays.

**Cell Culture and Transfection.** AC16 cell line were purchased from China Infrastructure of Cell Line Resource and were cultured in growth medium comprising high-glucose Dulbecco’s Modified Eagle medium (DMEM) supplemented with 10% fetal bovine serum (FBS), streptomycin (100 μg/ml) and penicillin (100 IU/ml). AC16 cell line were treated with PBS or Ang II (1 μM) for 48 hours to induce cardiomyocyte hypertrophy. HEK293 (human embryonic kidney) Cells were cultured in DMEM (H30022.01, HyClone, Connecticut, USA) with 10% FBS (Z7186FBS-500, ZETA LIFE, California, USA) and 1% penicillin-streptomycin. The mycoplasma contamination of the cell line was evaluated and the test results were negative.

**Transthoracic echocardiography.** Transthoracic echocardiography was performed in 9-month-old Bama minipigs using a GE Vivid E95 Color Doppler ultrasound device equipped with an S5-1 probe. Anesthesia was induced with intramuscular ketamine (15.5 mg/kg) and maintained with 1.5% isoflurane in 100% oxygen. Anesthesia depth was confirmed by absence of response to hindpaw pressure. Left ventricular ejection fraction (LVEF), left ventricular fractional shortening (LVFS) and other measured parameters were measured from M-mode images obtained from the parasternal short-axis or the parasternal short-axis view at the papillary muscle level. Pulsed-wave Doppler images in the apical four-chamber view and tissue Doppler images at the mitral annulus were acquired to evaluate diastolic function, including the early (E) and late (A) peak mitral inflow velocities, as well as the mean early diastolic mitral annular velocity (e’) obtained from the medial and lateral annuli (with an angle correction <20°).

**Quantitative real-time PCR.** Total RNA from pig cardiac tissues or AC16 cardiomyocytes was extracted for RT-qPCR, and the specific steps were performed as previously described [36]. The primer sequences for genes were listed in S1 Table.

**ChIP assay.** The ChIP assay was performed according to manufacturer’s protocol (ab500, Abcam, UK). In brief primary cells were first fixed in 1% formaldehyde and then quenched by 1.25 M glycine. Cells were sonicated to shear DNA into 200–500 bp fragments. The supernatant was collected and subjected to overnight immunoprecipitation with ZBED6 and IgG antibody at 4 °C in a rotary shaker, followed by reverse cross-link and protease K digestion. Genomic DNA–protein crosslinking complexes were collected, and purified DNA was subjected to qPCR. Primer sequences are listed in S1 Table.

**Western blotting.** The specific protein expression in pig cardiac tissue or AC16 cardiomyocytes was detected by following the steps previously described [37]. Antibodies used for protein detection are shown in S2 Table.

**Immunocytochemistry.** The cultured cells were fixed and washed as previously described [36]. The F-actin patterns were visualized using fluorescein isothiocyanate (FITC)-conjugated phalloidin (Yeasen, 40734ES75) for 30 min at room temperature, and DAPI (Solarbio, Beijing) nucleated for 3min. All images were captured on an inverted fluorescence microscope (BioTEK gen 5 Software). The cell size was quantified using Image J, and at least 50 cells per group were assessed.

**Seahorse analyzer.** Seahorse XF HS Mini analyzer (Agilent Technologies) was employed to evaluate the mitochondrial respiratory capacity by measuring the oxygen consumption rate (OCR) according to manufacturer instructions.

**Transmission electron microscopy.** After washing with PBS, cardiac tissue of pigs was fixed in 2.5% glutaraldehyde and 0.1 M sodium cacodylate buffer at 4°C overnight. All the samples were embedded and subjected to ultramicrotomy using LEICA EMUC7 (Leica microsystems, Vienna, Austria), Imaging was performed using a Transmission Electron Microscope (FEI TECNAI G2 SPIRIT BIO).

**Morphological analysis.** Fresh isolated ventricular septal myocardial tissues were fixed in 4% formaldehyde buffer (PFA) at 4 °C for 24 h, paraffin-embedded, and sectioned (5 μm). Sections were stained with H&E and Masson’s trichrome to assess morphology and ventricular fibrosis, and with WGA to measure cardiomyocyte cross-sectional area. Images were acquired using a fluorescence microscope (BioTEK gen 5 Software) and analyzed with ImageJ.

**Plasmid Construction.** The coding sequences of ZBED6 (NM_001174108.2) and MEOX1 (NM_004527.4) were cloned into pcDNA3.1 for overexpression. For human *ZBED6* knockout, sgRNA41: 5′-CCAGTTTCTTCACTCTCTCC-3′; sgRNA48: 5′-AGATGAAGAAGATGTGGTAG-3′ were inserted into pSpCas9(BB)-2A-Puro plasmid (Addgene, 62988) via BbsI. The MEOX1 promoter (pig/human) was cloned into pGL3-Basic (firefly luciferase; Promega), with pRL-TK (Renilla luciferase; Promega) as internal control. A mutated ZBED6-binding site (mutZBS) fragment was synthesized by Qingke Biotechnology (Wuhan, China). The si-MEOX1, si-BDNF and si-MCF2L2 were synthesized from GenePharma (Shanghai, PRC). The MEOX1 siRNA sequences as listed in S3 Table.

**Plasmid transfection and luciferase reporter assay.** 293T cells in 12-well plates were co-transfected with pig MEOX1 promoter luciferase reporters (WT/mutated ZBED6-binding site, 500ng), pcDNA3.1-ZBED6, and PRLTK (50 ng) vector using Lipofectamine 3000 (Invitrogen) for 48h. To assess human ZBED6-mediated regulation of MEOX1, AC16 and ZBED6-deficient AC16 cells were transfected with human MEOX1 promoter and PRLTK vector, then lysed for reporter activity measurement using Dual-luciferase Reporter Assay System (E1980, Promega, Wisconsin, USA).

**Statistical Analysis.** Data are presented as means ± standard error of the mean of independent experiments. The Shapiro-Wilk normality test was used for the normal distribution test before analysis. The parametric statistics were used for normal distribution data. The unpaired 2-tailed Student *t* test or 2-way ANOVA with the Duncan post hoc test was used for comparison between 2 or multiple groups. Statistical correlation was analyzed using Pearson’s Correlation Coefficient (two-tailed, confidence interval (CI)  =  95%). Statistical analysis was performed using GraphPad Prism version 8.0.2 for Windows (GraphPad Software, USA, www.graphpad.com). P  <  0.05 was considered significant. Representative images/figures were chosen according to the approximation of the mean/average of the indicated group.

## Supporting information

S1 Fig ZBED6 deficiency leads to the diastolic dysfunction in pig hearts and cardiomyocytes. (A) Western blot analysis of ZBED6 protein levels in hearts from *ZBED6* knockout pigs and their wild type littermates (n = 3). (B and C) Average fractional shortening and average velocities in cardiomyocytes from WT and *ZBED6*^-/-^ pigs. (D) Contraction time, (E) relaxation time, (F) fractional shortening and (G) resting sarcomere length (SL) are shown (N = 3 pigs, n = 50–60 myocytes per group). (H-M) Echo parameters of *ZBED6*^−/−^ and WT pigs (n = 4). LVESV: left ventricular end-systolic volume, LVEDV: LV end diastolic volume, LVIDd: LV internal diameter at end diastole, LVIDs: LV internal diameter at end systole, LVPWd: LV posterior wall thickness in diastole, LVPWs: LV end-systolic posterior wall thickness (n = 4). Data are presented as mean ± SEM. *P < 0.05, **P < 0.01.
(DOCX)

S2 Fig Loss of *ZBED6* upregulates hypertrophic markers in human cardiomyocytes. (**A** and **B**) *ZBED6*-KO cardiomyocytes were generated using CRISPR/Cas9, knockout efficiency confirmed by second-generation sequencing and PCR. (**C**) Relative mRNA levels of *ANP*, *BNP* and *MYH7* were analyzed by RT-qPCR in WT and *ZBED6*-deficient AC16 cells treatment with vehicle or Ang II (n = 3). (**D** and **E**) Immunoblotting and quantitation of ANP protein levels in each group (n = 3). Data are expressed as mean ± SEM; * *P* < 0.05, ** *P* < 0.01.
(DOCX)

S3 Fig *ZBED6* knockout induces mild fibrosis in pigs. (A) Relative mRNA levels of *ANP*, *BNP* and *MYH7* in cardiac tissues from WT and *ZBED6*-deficient pigs (n = 3). (B) Western blot analysis of ANP in cardiac tissue in each group (n = 3). (C and D) Representative H&E and Masson’s trichrome staining of cardiac tissue from 9-month-old *ZBED6*^*−/−*^ and WT pigs. Scale bar, 50 μm. (E) Quantitation of the fibrotic area (%) (n = 3). (F) *α-SMA* mRNA expression levels in WT and *ZBED6* knockout pigs (n = 3). Data are expressed as mean ± SEM; * *P* < 0.05, ns, not significant.
(DOCX)

S4 Fig Mitochondrial structure and function are preserved in ZBED6-Deficient pigs and AC16 cells. (A) Representative electron micrographs showing mitochondrial morphology in left ventricular tissue from WT and *ZBED6*-deficient pigs. Scale bar, 2 μm. (B) Protein expression levels of respiratory chain components in cardiac tissues from WT and *ZBED6*-deficient pigs (n = 3). (C) Oxygen consumption rate (OCR) in WT and *ZBED6*-deficient AC16 cells (n = 3). (D and E) The mRNA expression levels of mitochondrial marker genes in *ZBED6*-deficient pig cardiac tissues (n = 4) and *ZBED6*-deficient AC16 cells compared with their control (n = 3). Data are expressed as mean ± SEM; * *P* < 0.05, ** *P* < 0.01, ns, not significant.
(DOCX)

S5 Fig ZBED6 deficiency promotes cell apoptosis in pig hearts and human cardiomyocytes. (A and B) Representative immunofluorescence images of TUNEL staining in hearts from WT and ZBED6-deficient pig (n = 6). Scale bars, 100 μm. (C and D) Representative immunofluorescence images of TUNEL staining in ZBED6-deficient or control AC16 cells (n = 6). Scale bars, 300 μm. Data are expressed as mean ± SEM; * *P* < 0.05, ** *P* < 0.01, ns, not significant.
(DOCX)

S6 Fig Identification and functional validation of conserved ZBED6 target genes in cardiomyocytes. (A) Bubble plot analysis the 18 genes expression trend in pig and human transcriptome datasets. (B) Luciferase reporter assays in WT or *ZBED6*-OE 293T cells transfected with *MEOX1, BDNF*, *MCF2L2* and *ARHGAP28* promoter constructs. (C) The knockdown efficiency of *BDNF* (left) and *MCF2L2* (right) in AC16 cells treated with si*BDNF* or si*MCF2L2* (n = 3). (D and E) Representative immunofluorescence images of α-actinin (green) and DAPI (blue) in WT and *ZBED6*-deficient cells transfected with si*BDNF* or si*MCF2L2* for 48 hours. Scale bar, 25 μm. Data are expressed as mean ± SEM; * *P* < 0.05, ** *P* < 0.01, ns, not significant.
(DOCX)

S7 Fig ZBED6 controls the transcription of a cardiac hypertrophy regulator MEOX1. (A) Relative mRNA expression level of *MEOX1* in WT and *ZBED6*-depletion cardiomyocytes (n = 3). (B and C) The protein level of MEOX1 in *ZBED6*-knockout porcine hearts and human cardiomyocytes compared with controls (n = 3). (D) MEOX1 protein level in cardiac tissue from healthy individuals (n = 6) and cardiac hypertrophy patients (n = 5). (E) Schematic of wild-type and ZBS-mutant sequences. (F) Luciferase activity of wild-type (ZBS-LUC) or mutant (mZBS-LUC) *MEOX1* reporters in the presence of ZBED6 (n = 6). (G) ChIP assay analysis of ZBED6 binding to the *MEOX1* promoter region in pig cardiac tissue (n = 3). (H) The relative luciferase activity of the *MEOX1*-ZBS-LUC reporter in *ZBED6*-deficient and control cells (n = 12). Data are expressed as mean ± SEM; * *P* < 0.05, ** *P* < 0.01.
(DOCX)

S8 Fig ZBED6 deficiency-induced cardiomyocyte hypertrophy is independent of IGF2. (A) Relative *IGF2* mRNA levels in each group (n = 3). (B) The protein levels of IGF2, p-ERK1/2, and total ERK1/2 in each group (n = 3). (C) The quantization of IGF2 protein levels in each group (n = 3). (D and E) Representative immunofluorescence images of α-actinin and quantification of cell size in ZBED6-deficient AC16 cells treated with si-*IGF2* or si-*Control* for 48 hours (n = 50). Scale bars, 25 μm. Data are presented as mean ± SEM. *P < 0.05, **P < 0.01, ns, not significant.
(DOCX)

S1 Raw Gels Uncropped western blots. (PDF)

S1 Table Primers for RT-PCR analysis. (DOCX)

S2 Table Antibodies information. (DOCX)

S3 Table siRNA sequences. (DOCX)

S1 Data Raw Data. (XLSX)
