# Supplementary material for: ZBED6 deficiency drives pathological cardiac hypertrophy through MEOX1–ERK1/2–dependent autophagy dysfunction
Source: PLoS Genet. 2026 Oct 8;22(10):e1012329. doi: 10.1371/journal.pgen.1012329 (PMC13648969; doi:10.1371/journal.pgen.1012329)

Fig 1A

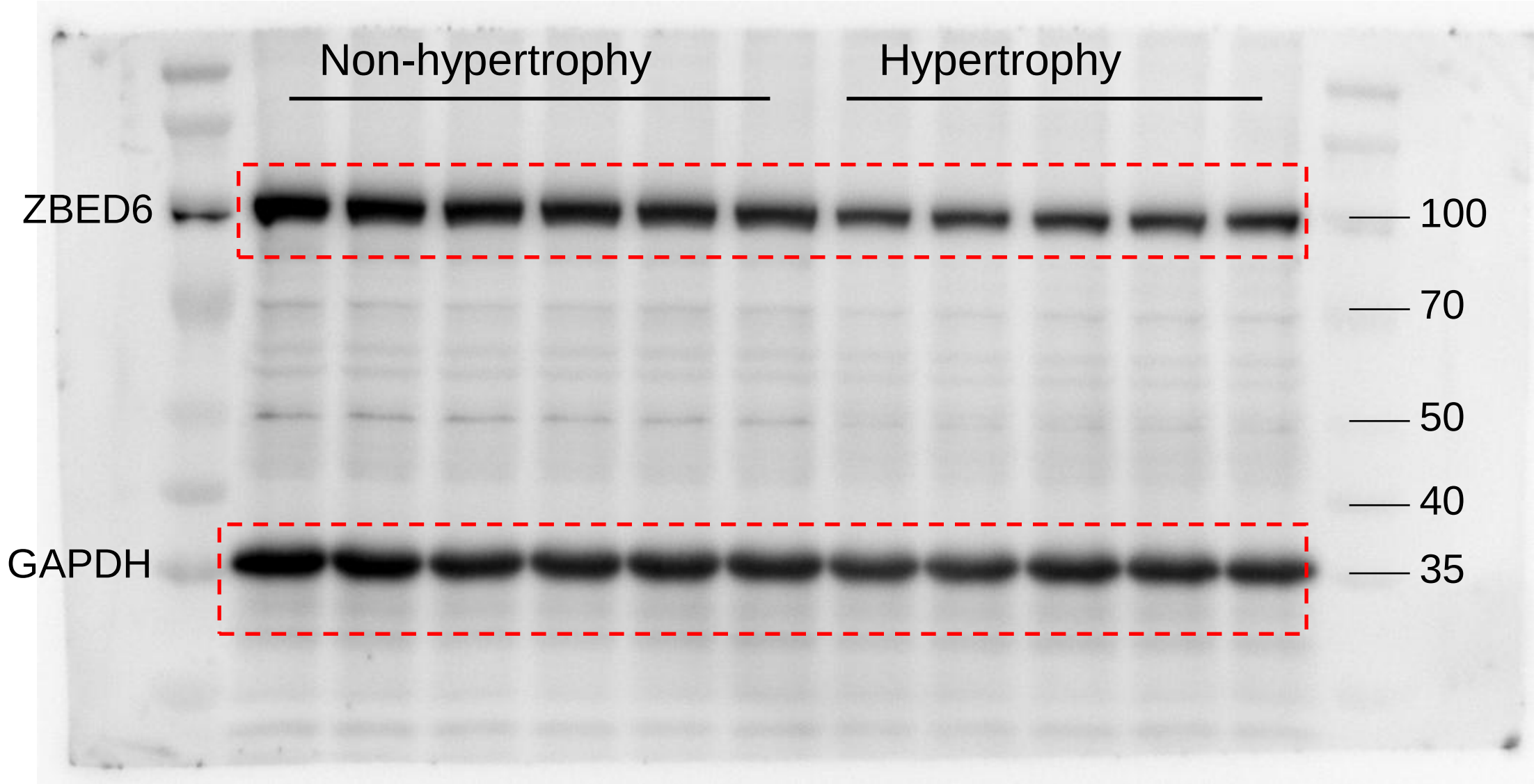

Non-hypertrophy
Hypertrophy
ZBED6
GAPDH
100
70
50
40
35

Fig 1E

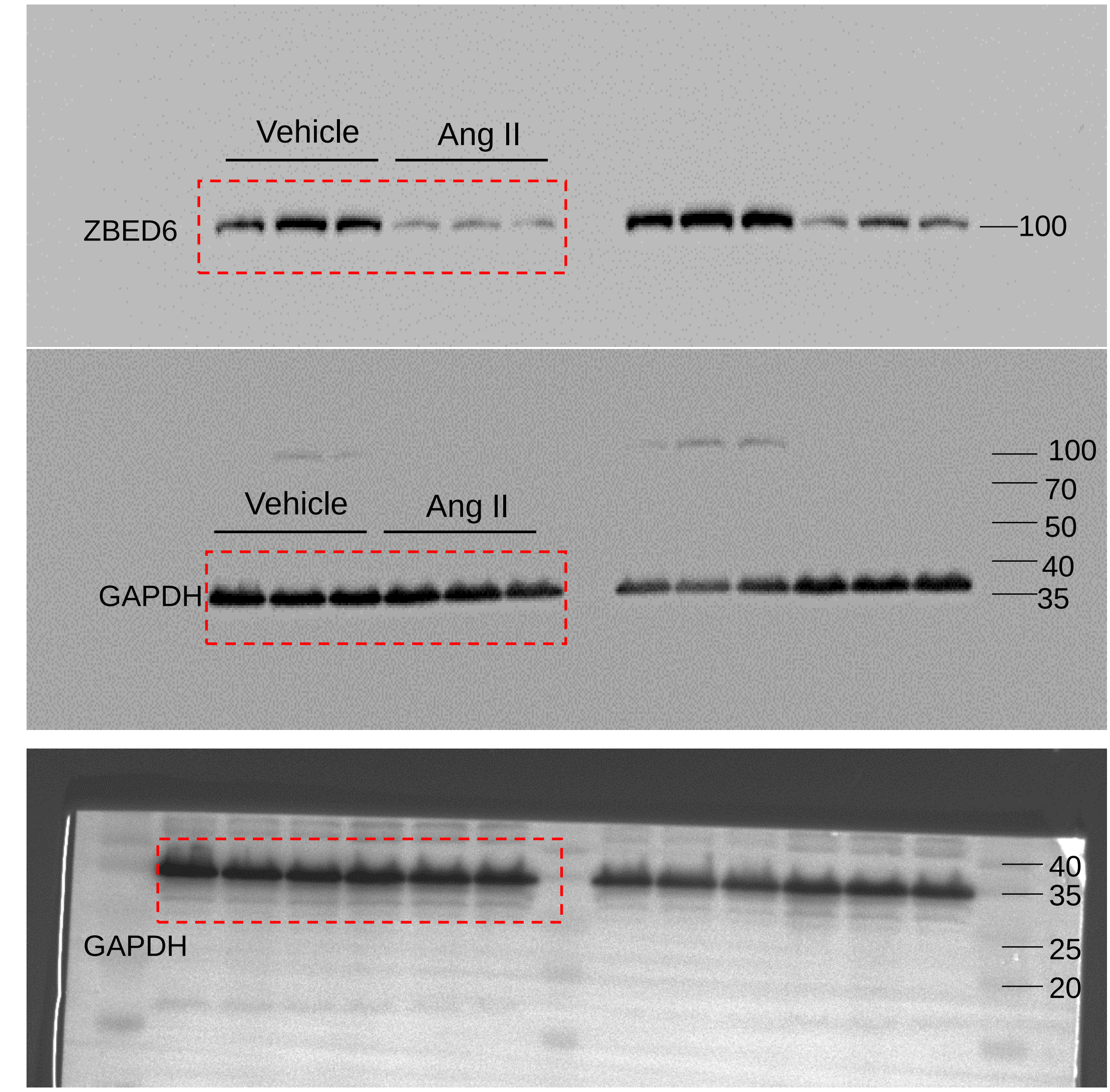

Vehicle
Ang II
ZBED6
100
Vehicle
Ang II
GAPDH
100
70
50
40
35
GAPDH
40
35
25
20

Fig 3B

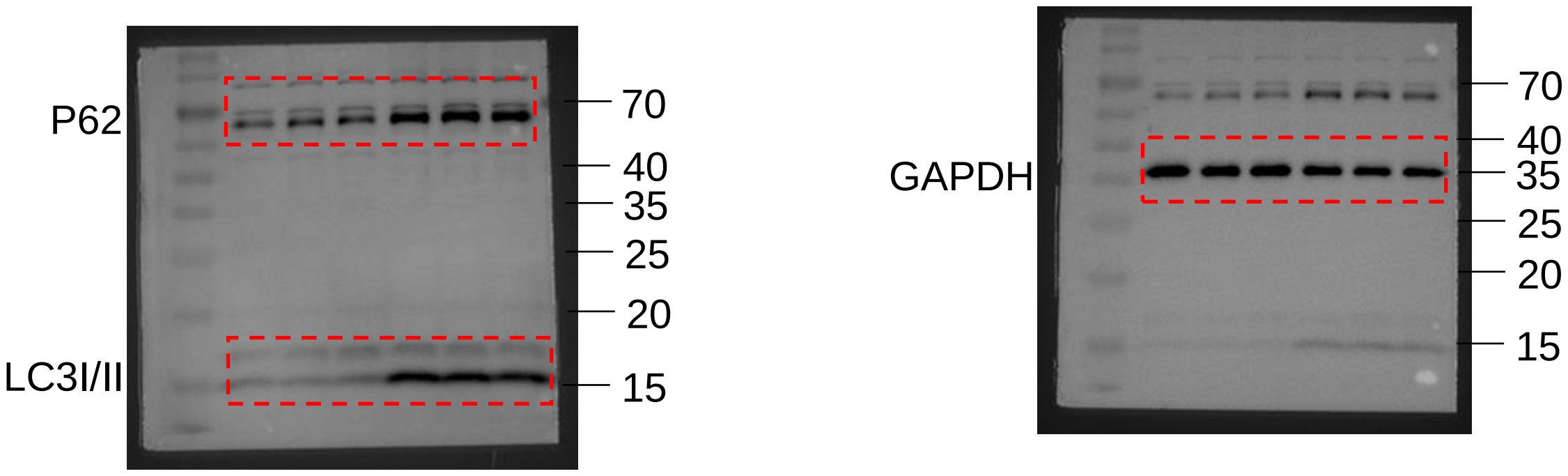

Fig 3C

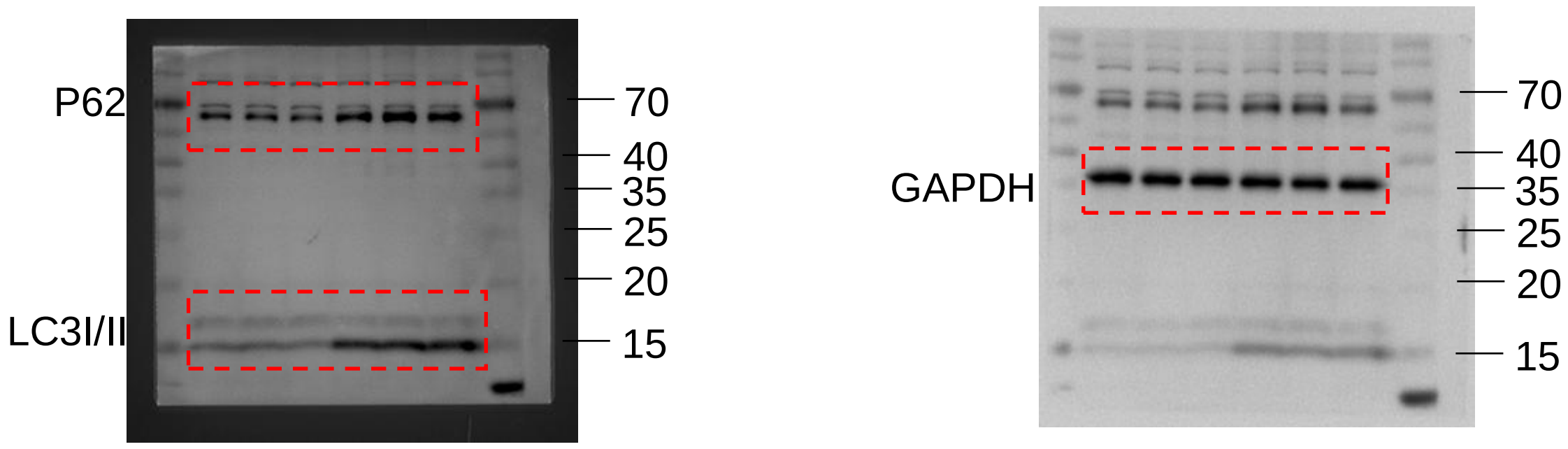

Fig 3E

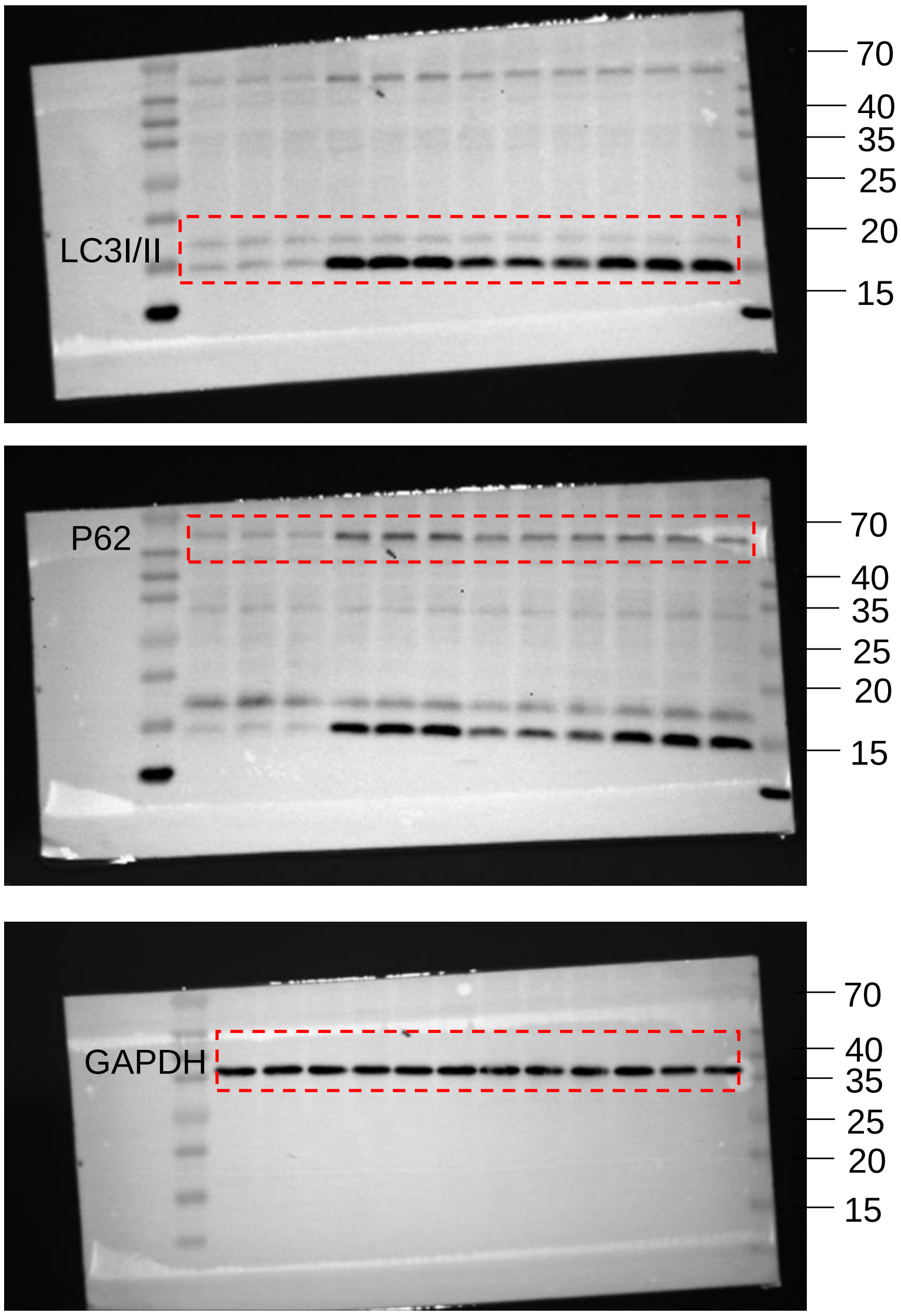

Fig 4F

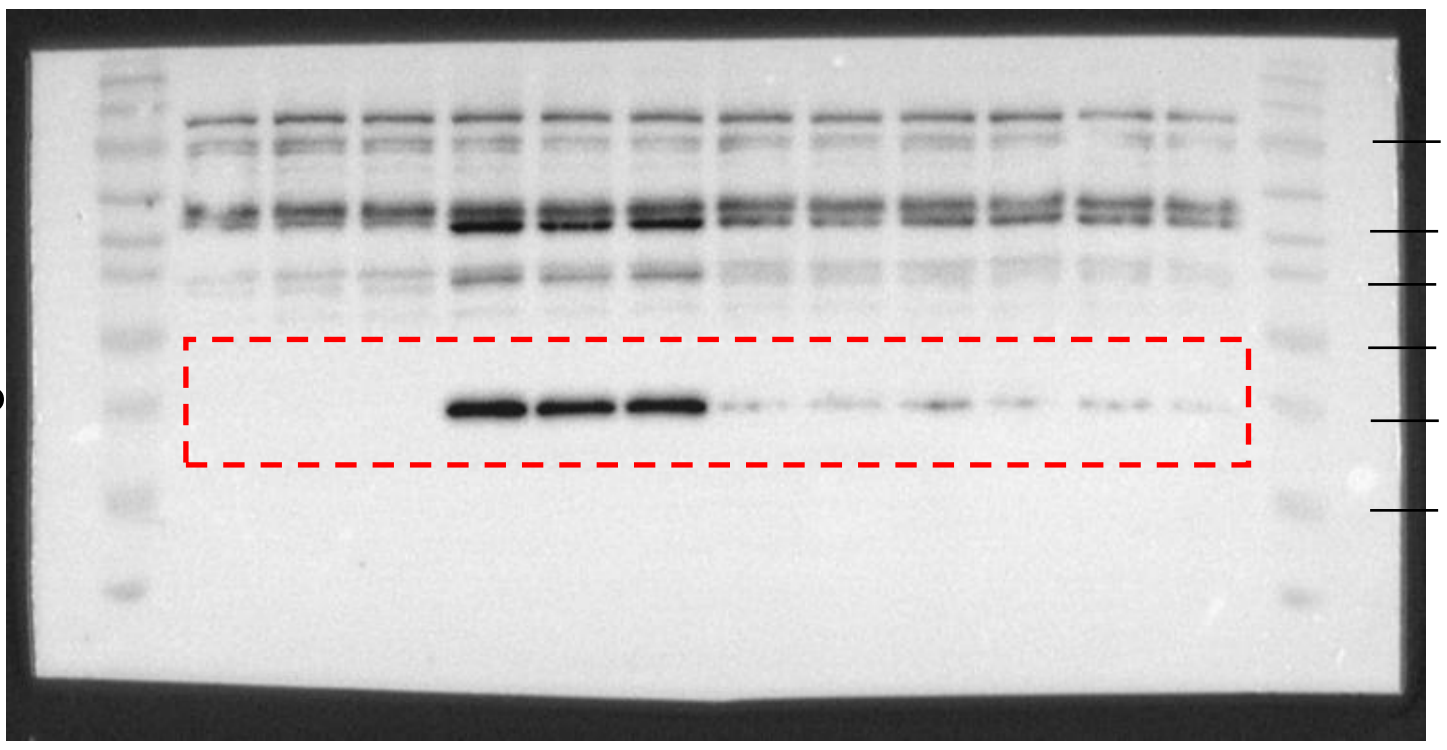
ANP
70
40
35
25
20
15

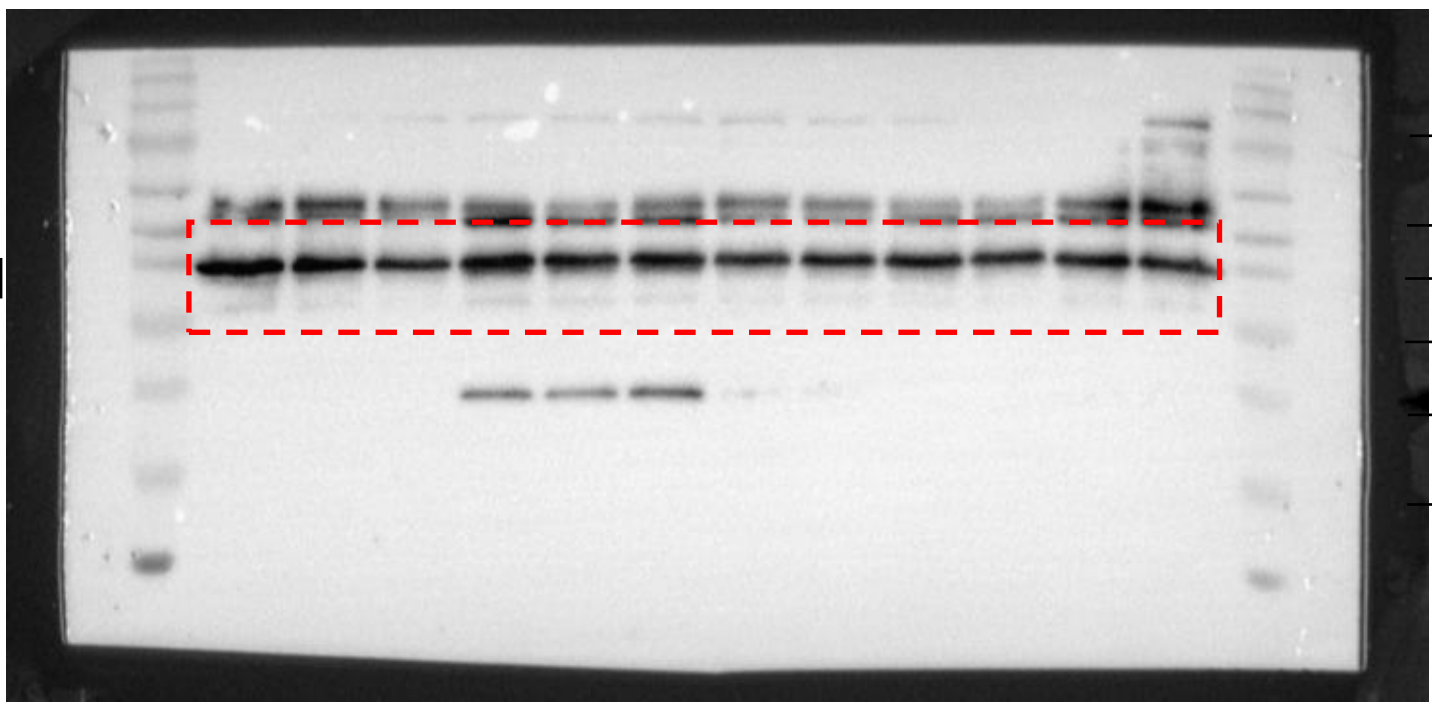
GAPDH
70
40
35
25
20
15

Fig 4H

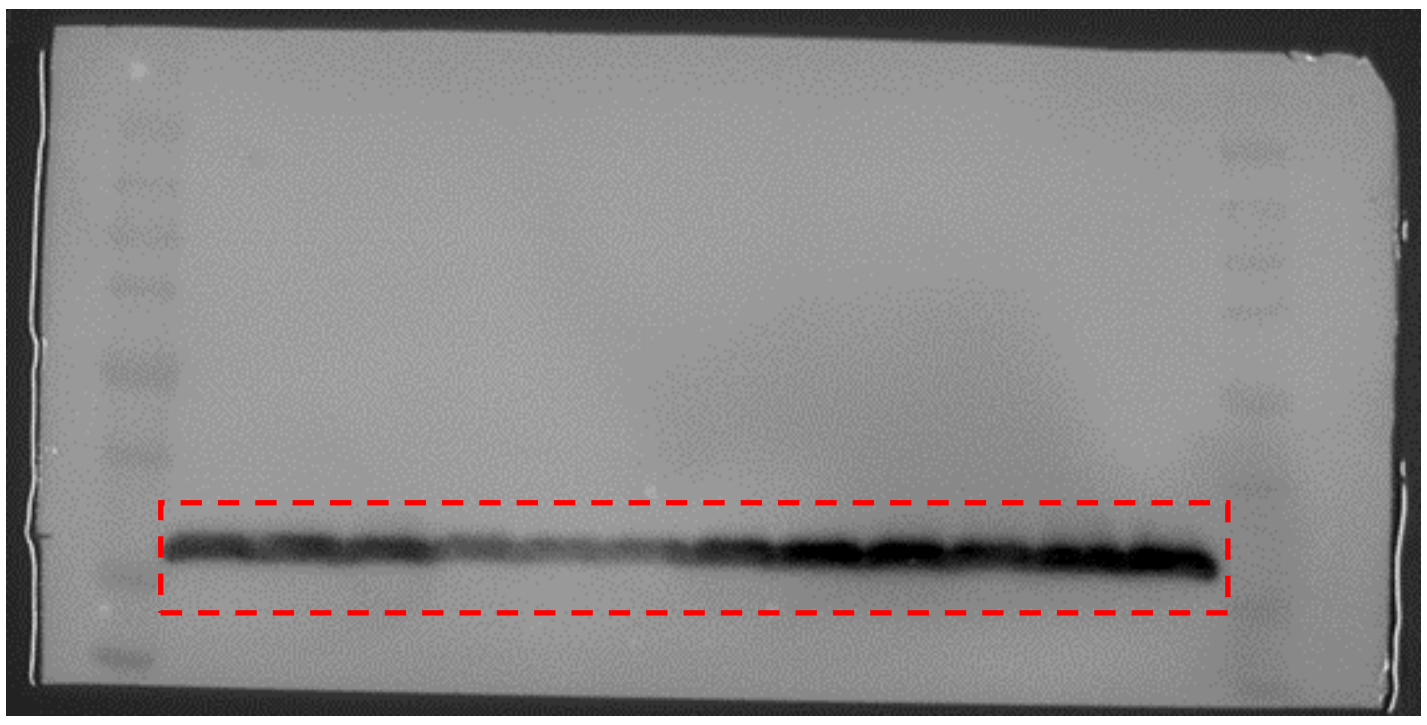
ANP
70
40
35
25
20
15

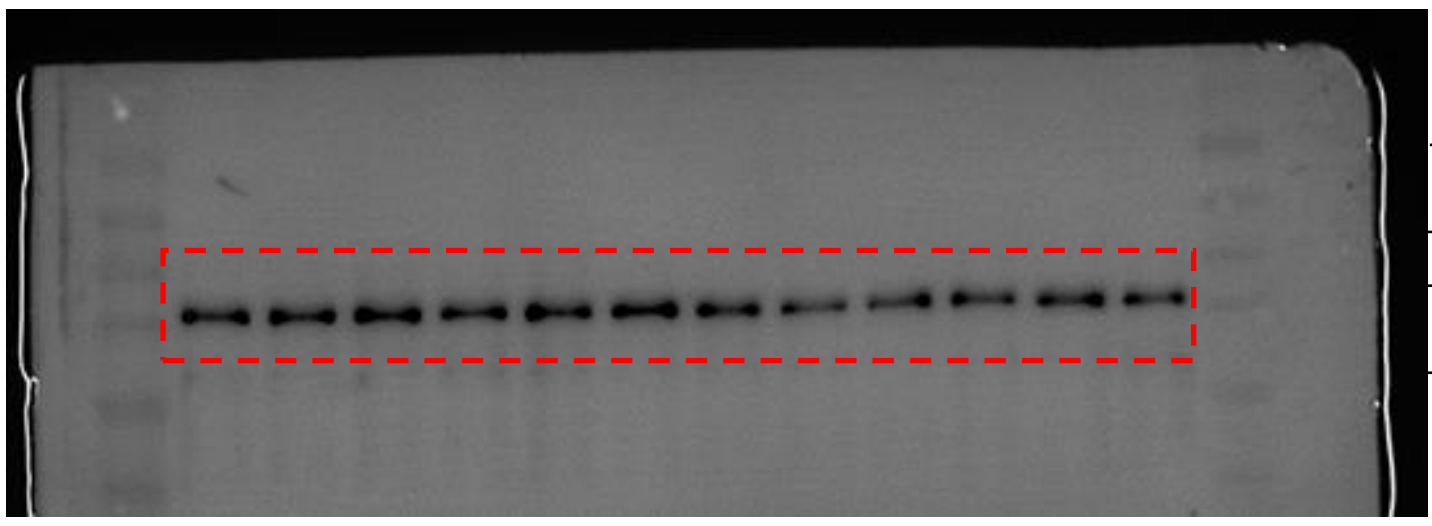
GAPDH
70
40
35
25

Fig 5B

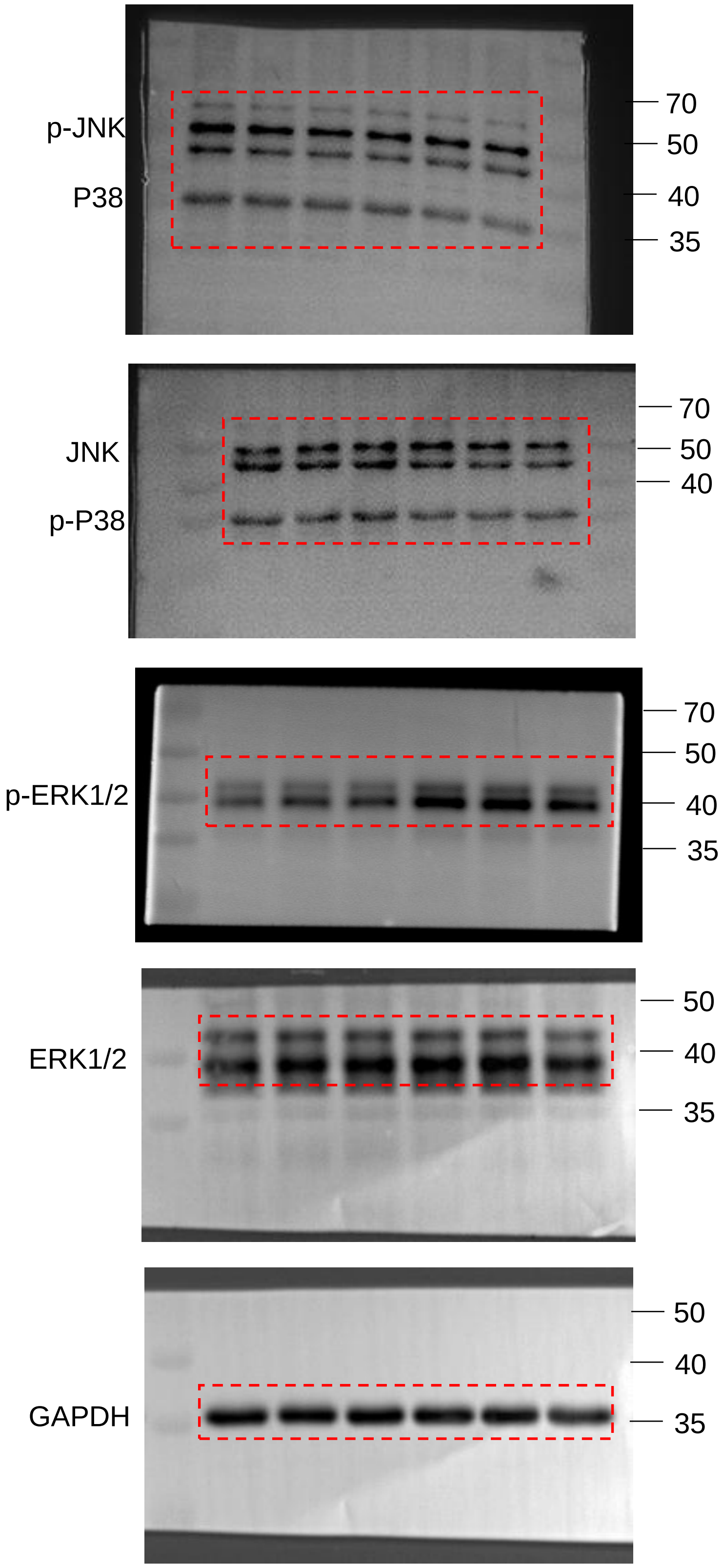
p-JNK
P38
70
50
40
35
JNK
p-P38
70
50
40
p-ERK1/2
70
50
40
35
ERK1/2
50
40
35
GAPDH
50
40
35

Fig 5C

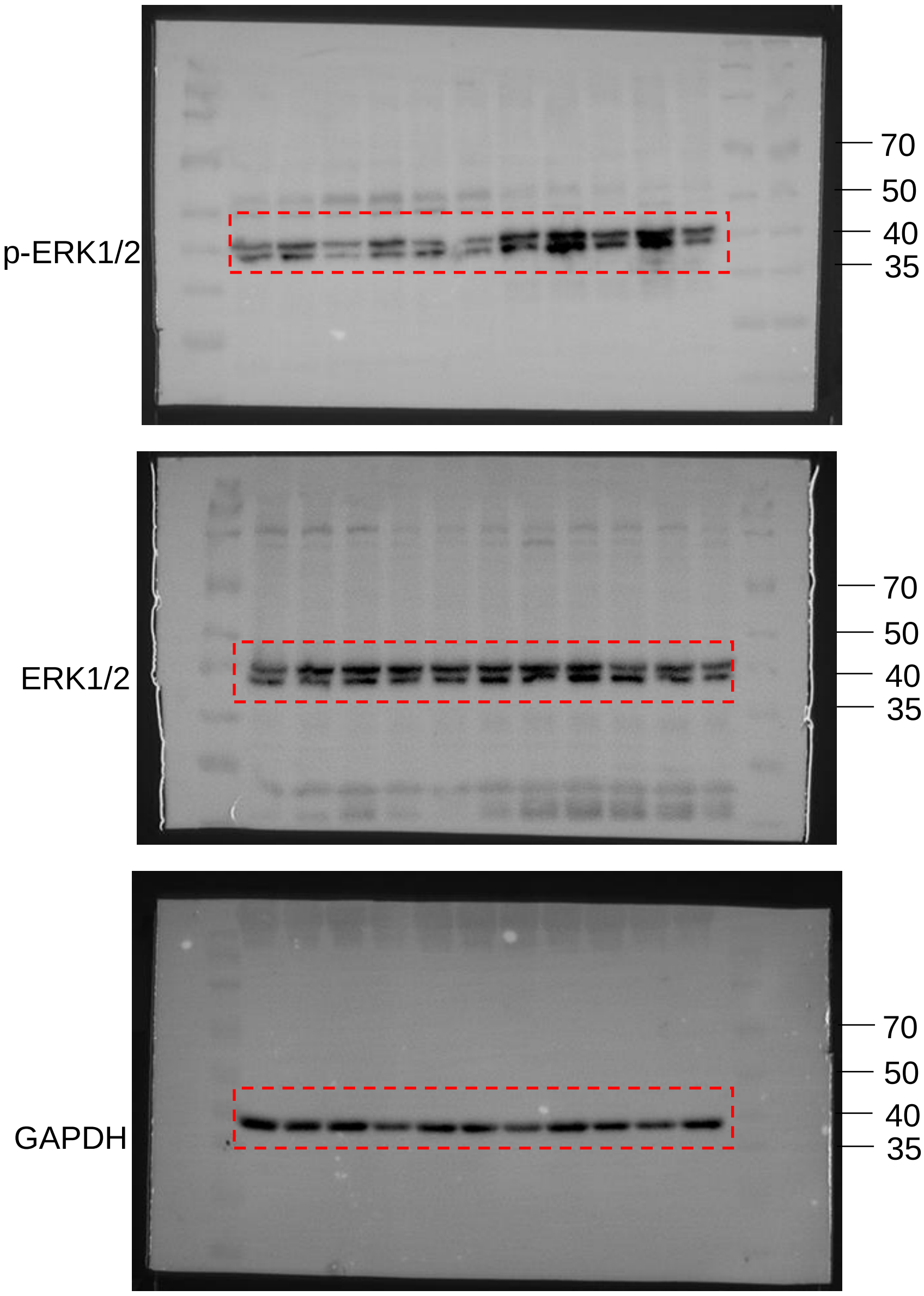

Fig 5D

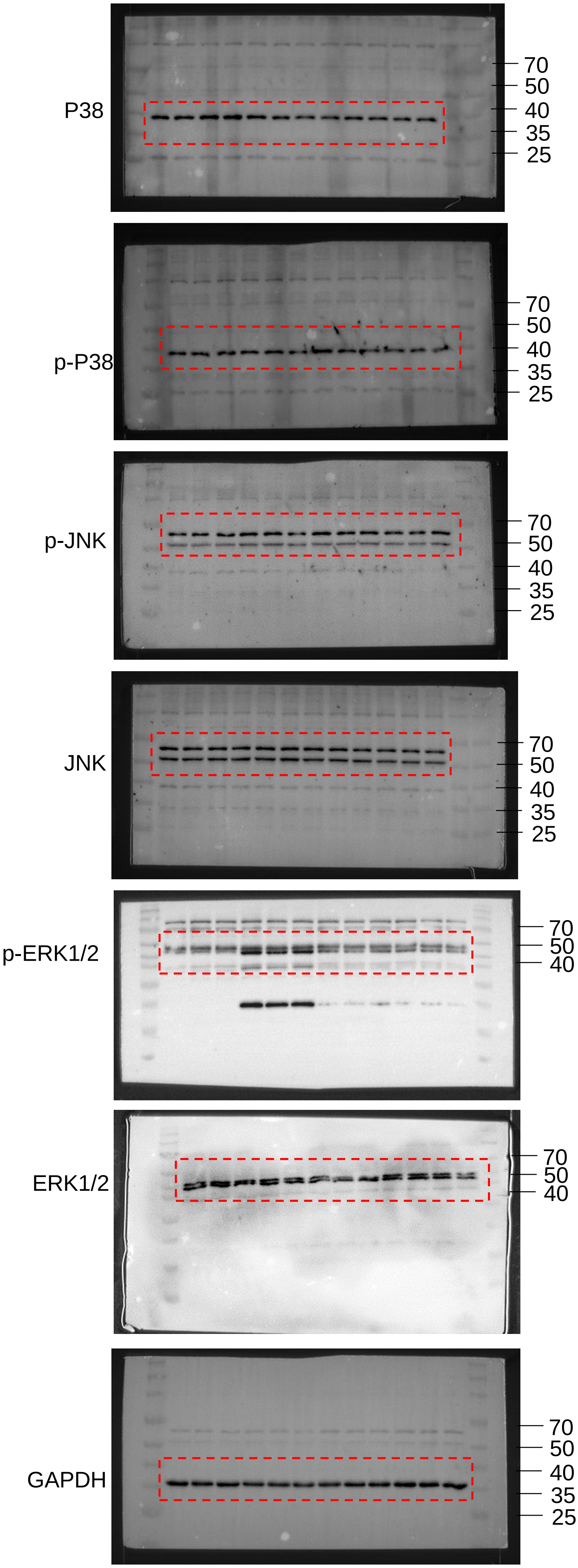
P38
70
50
40
35
25
p-P38
70
50
40
35
25
p-JNK
70
50
40
35
25
JNK
70
50
40
35
25
p-ERK1/2
70
50
40
ERK1/2
70
50
40
GAPDH
70
50
40
35
25

Fig 5E

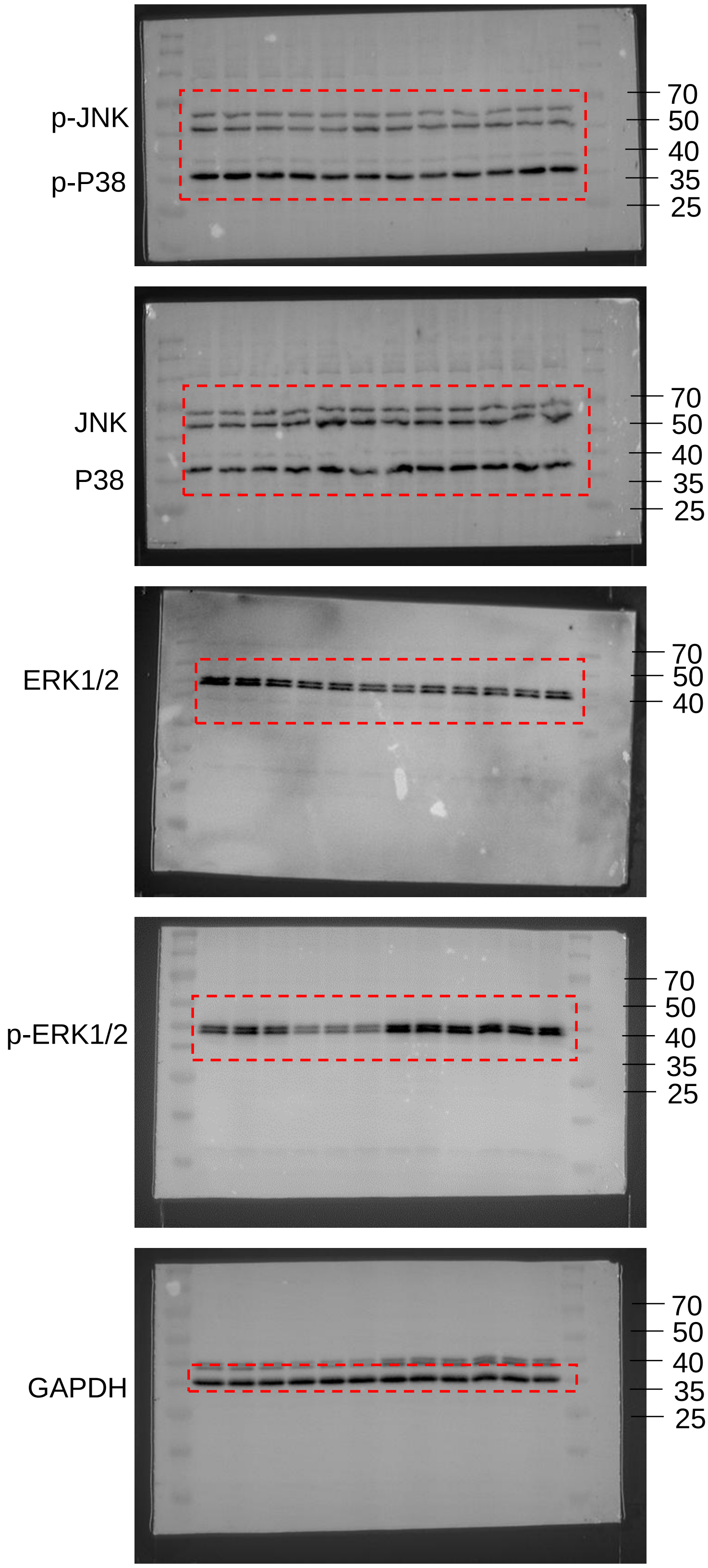

p-JNK
p-P38
70
50
40
35
25
JNK
P38
70
50
40
35
25
ERK1/2
70
50
40
p-ERK1/2
70
50
40
35
25
GAPDH
70
50
40
35
25

Fig 6E

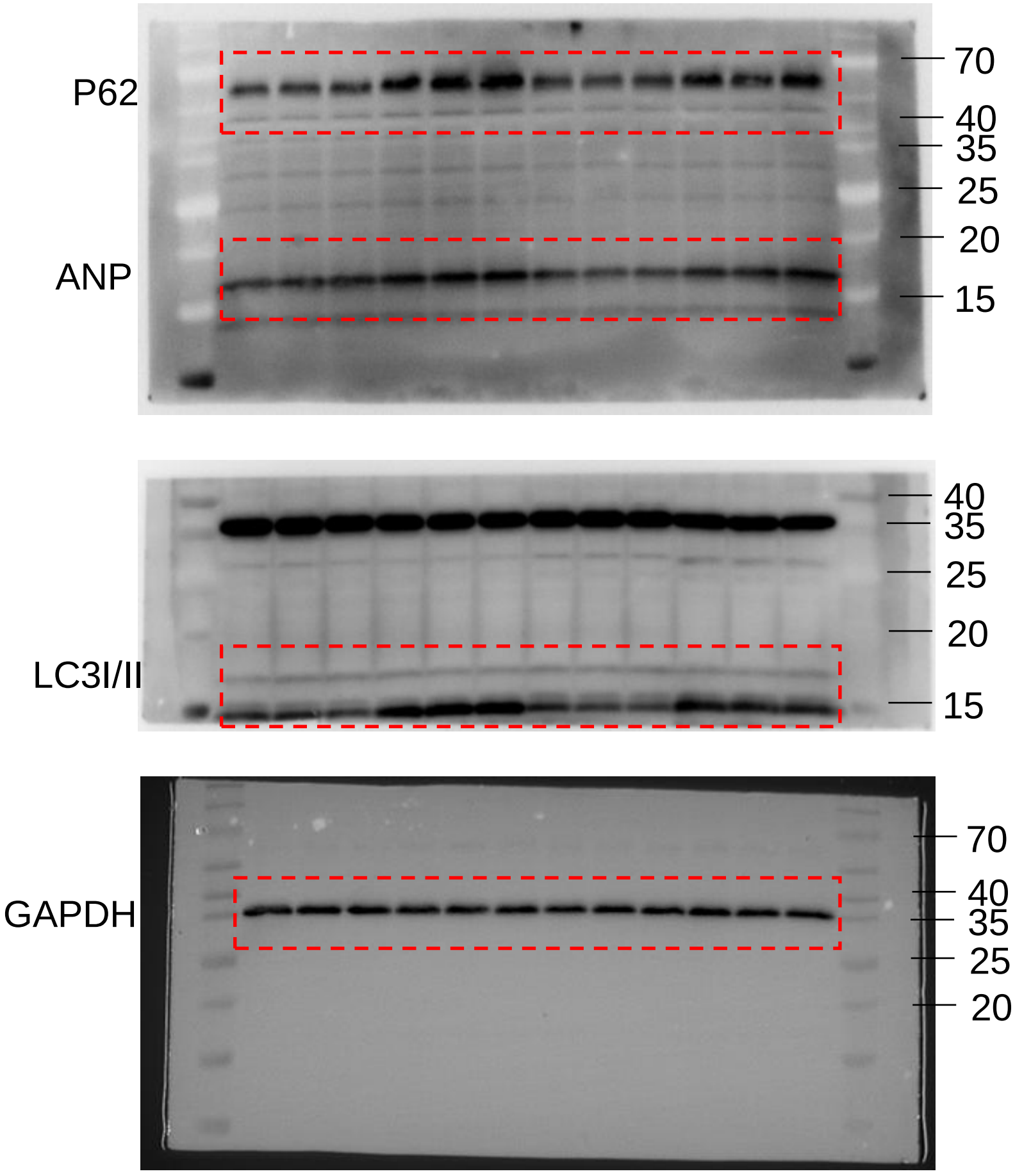

S1 Fig 1A

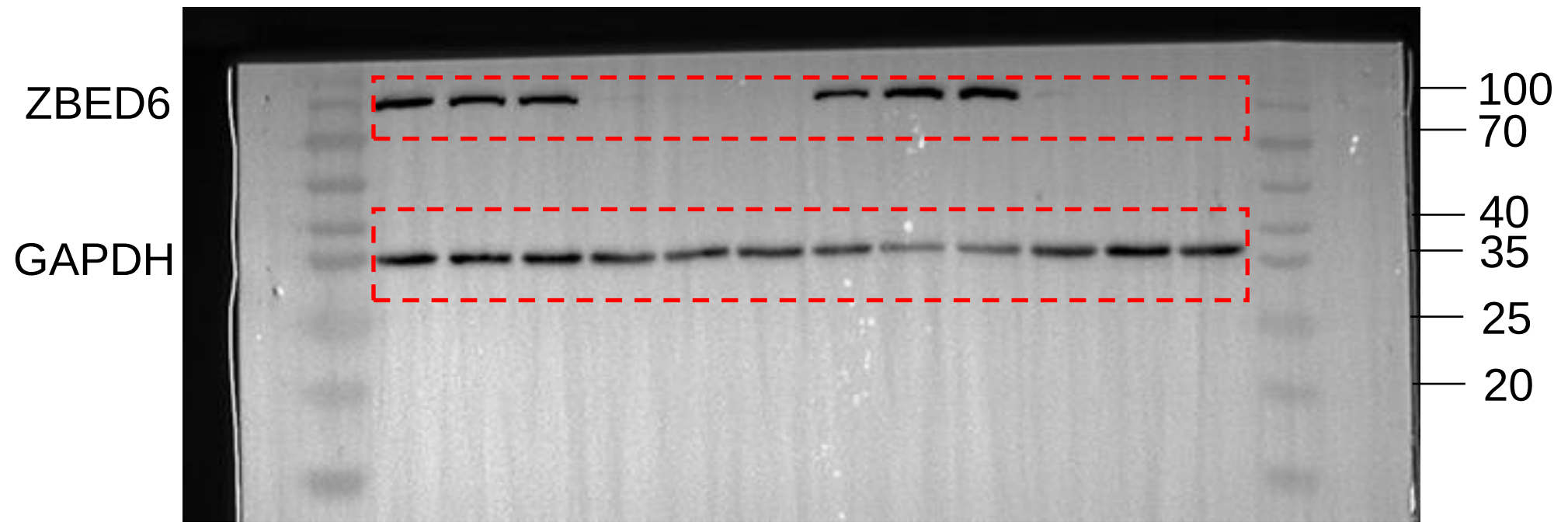

S2 Fig 2B

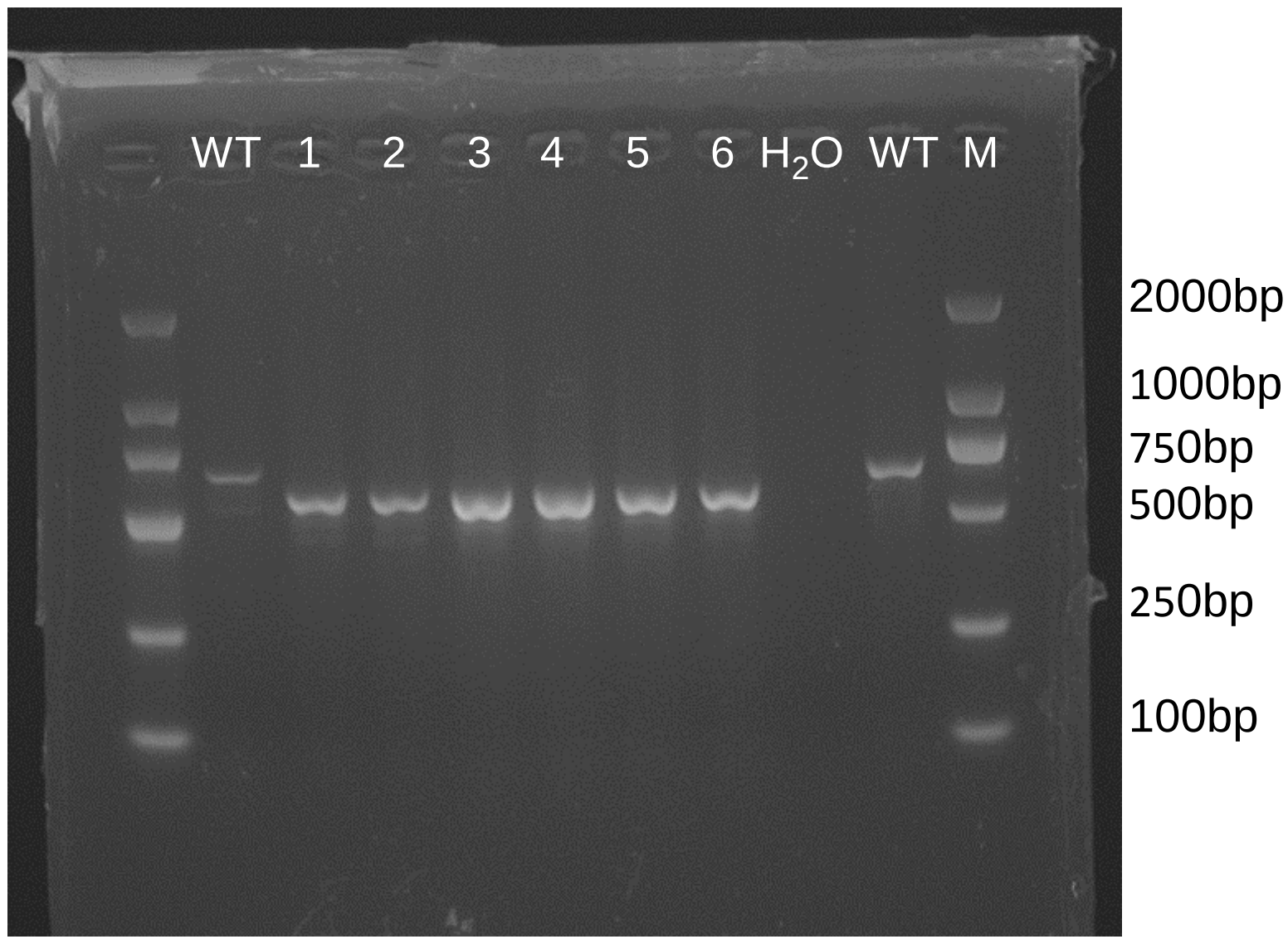

S2 Fig 2D

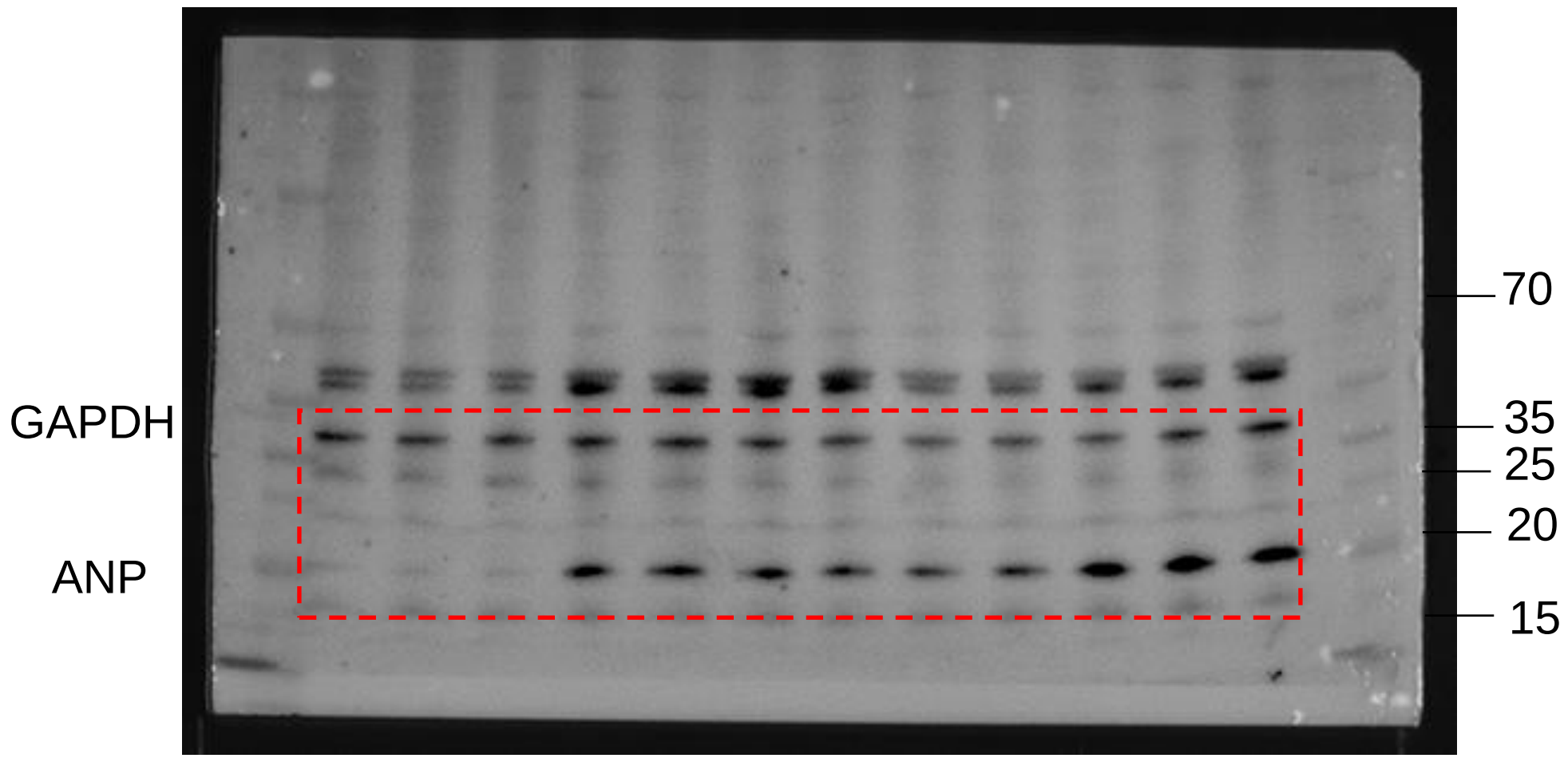

S3 Fig 3B

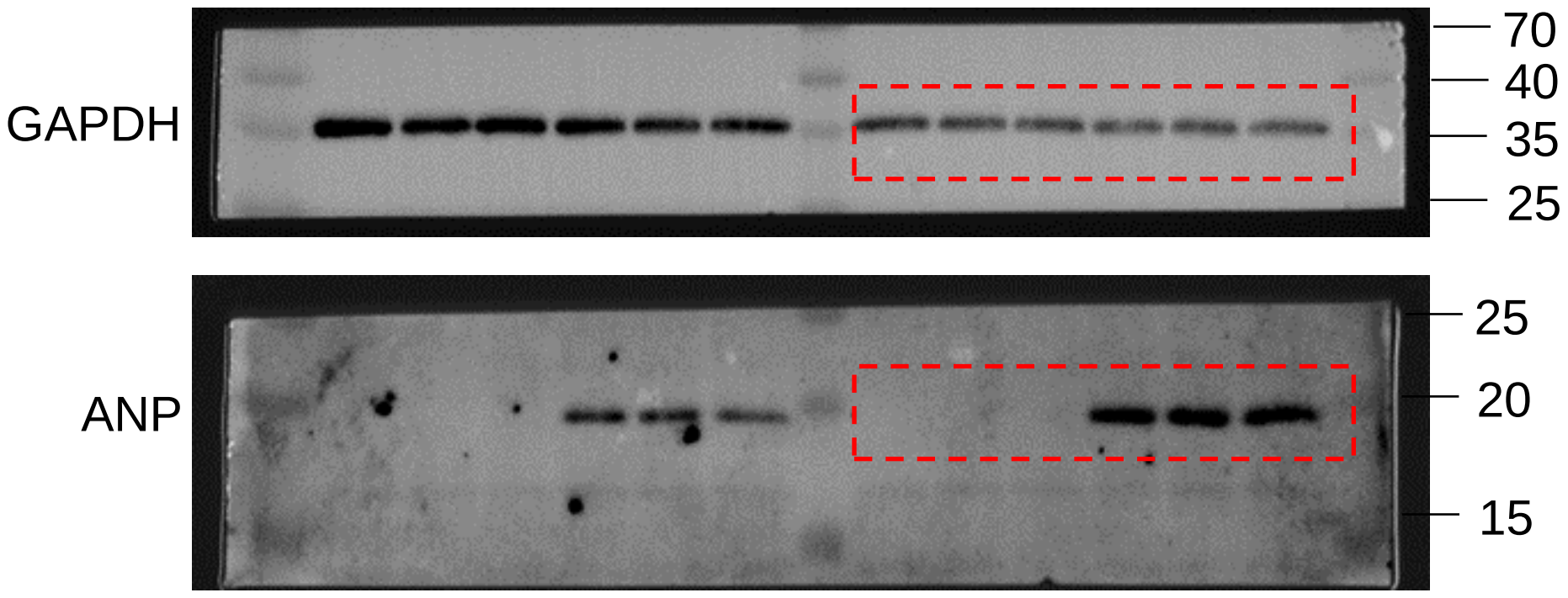

S4 Fig 4B

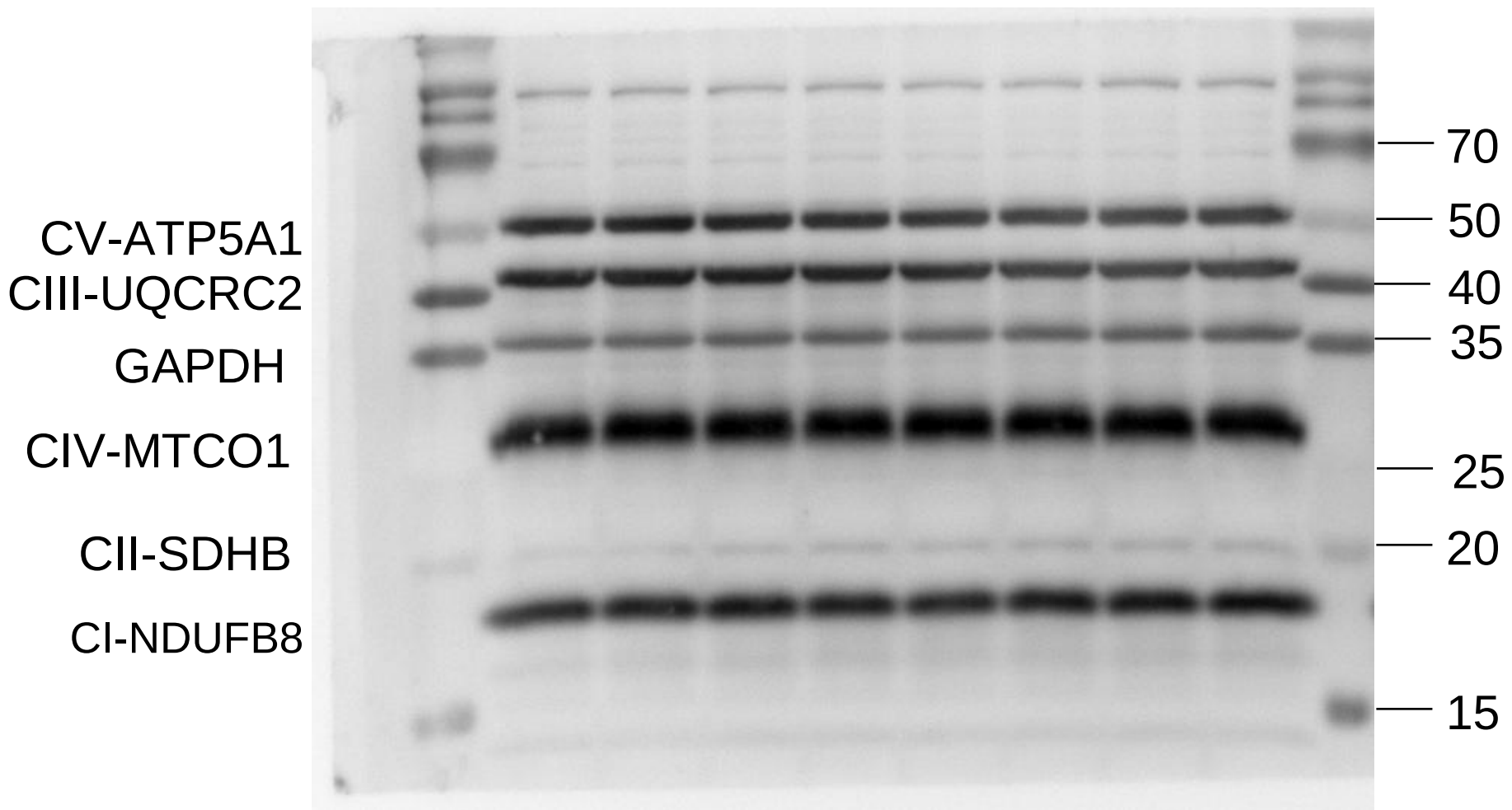

S7 Fig 7B

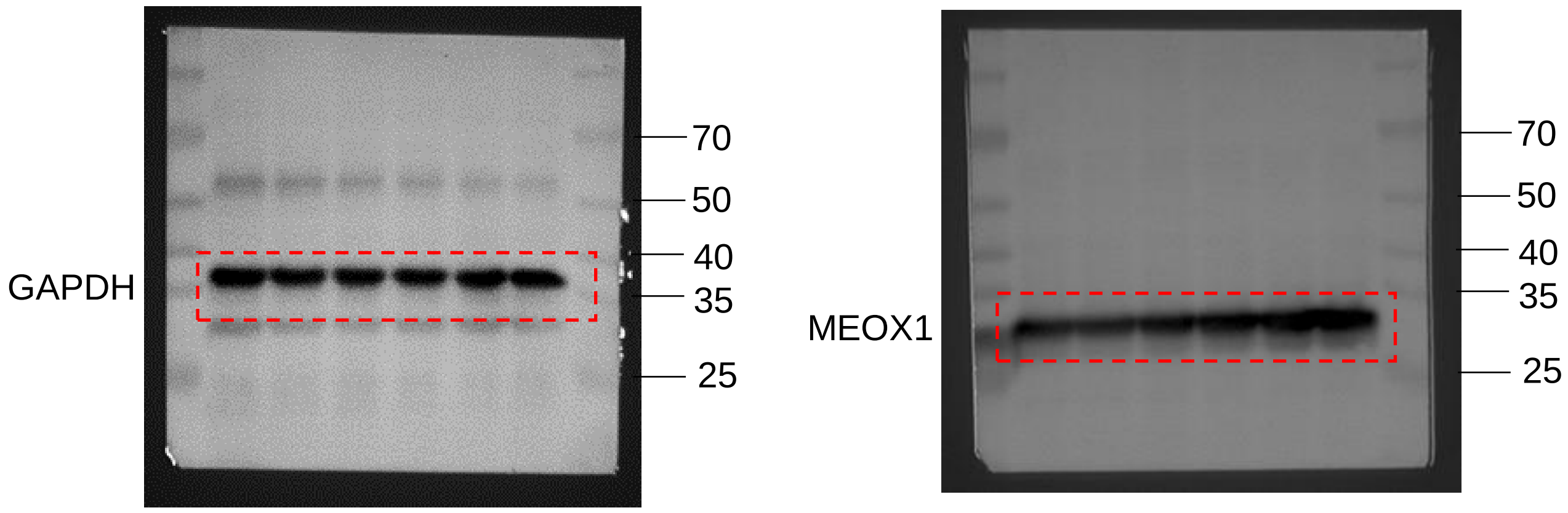

S7 Fig 7C

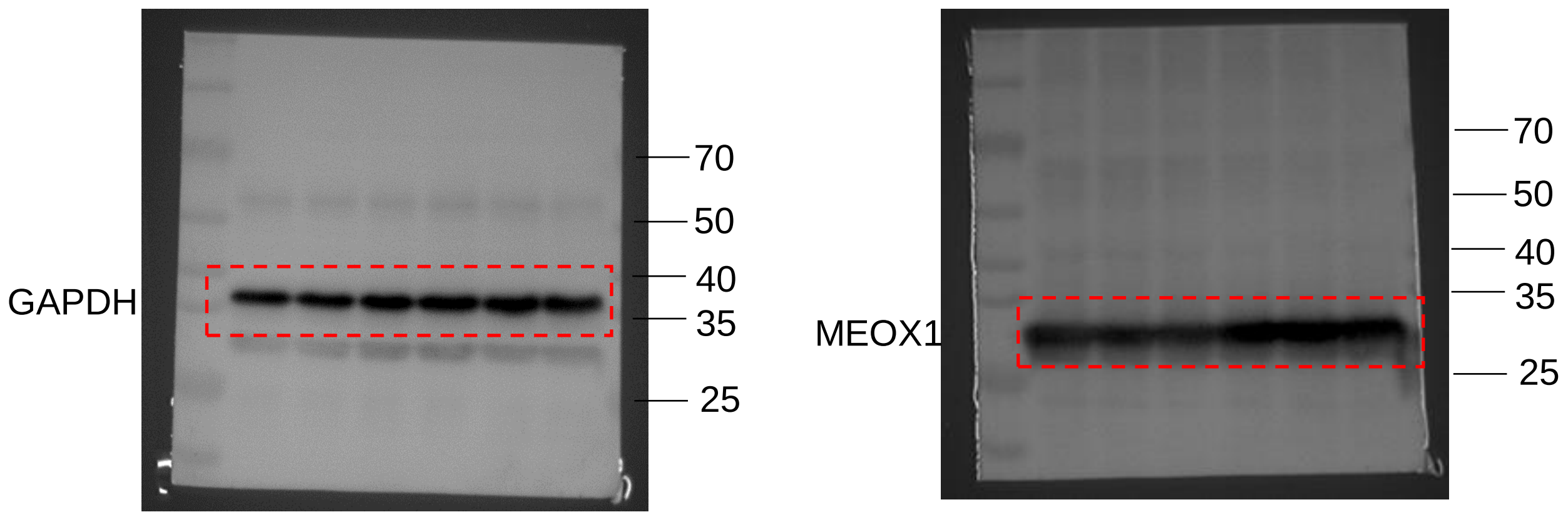

S7 Fig 7D

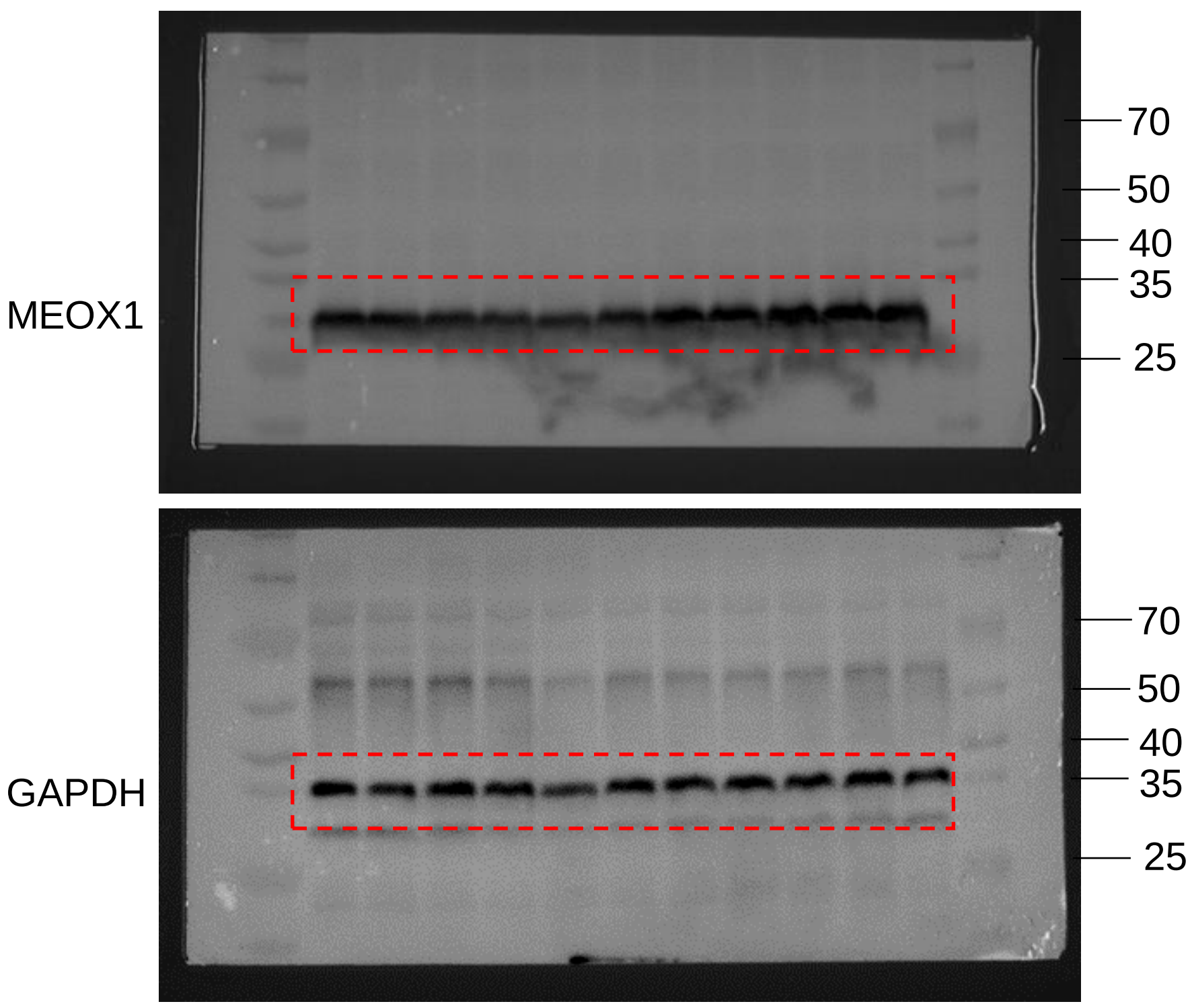

S8 Fig 8B

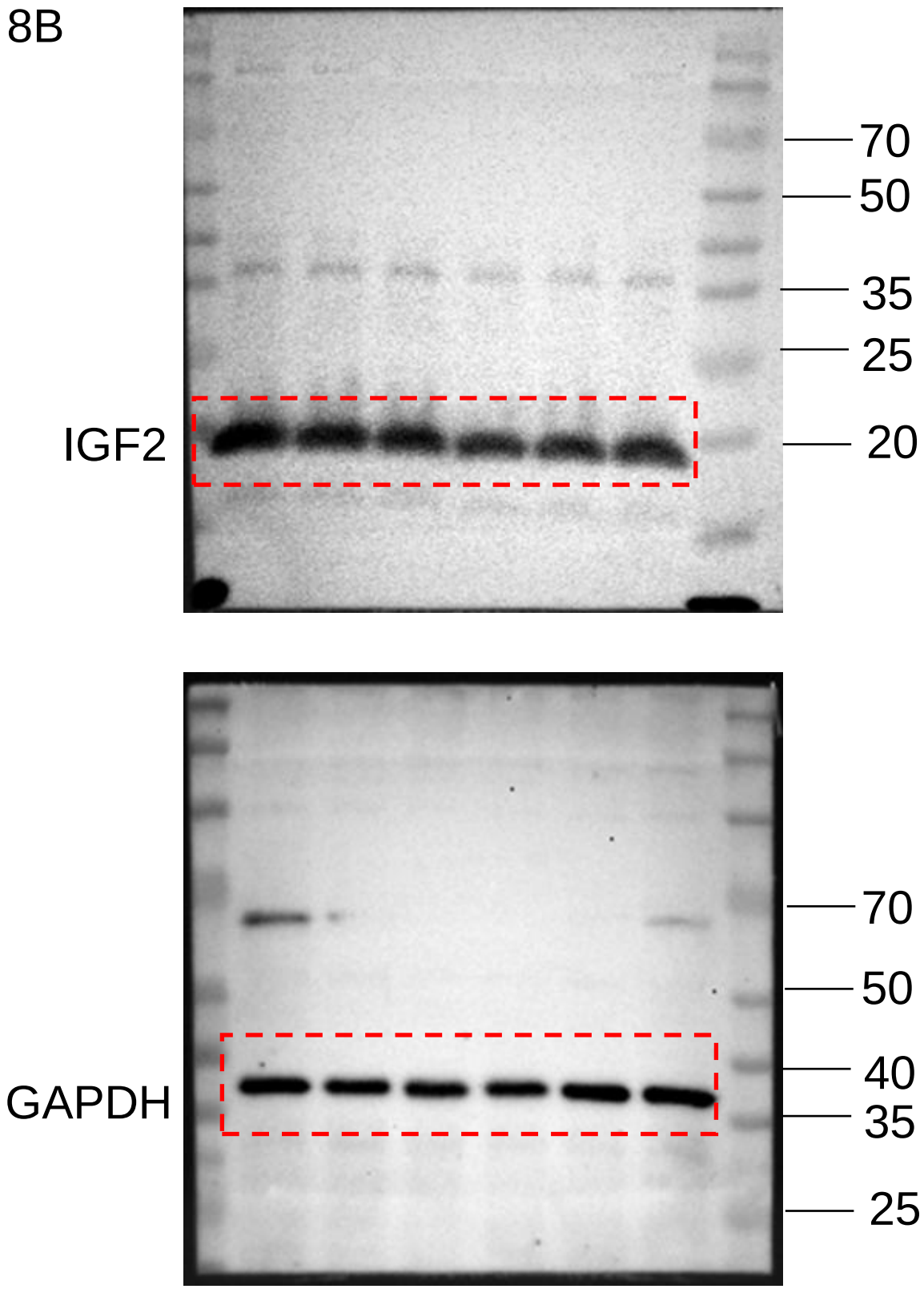

Supplement: Supplementary file 1 [file pgen.1012329.s009.pdf]
