# Supplementary material for: ZBED6 deficiency drives pathological cardiac hypertrophy through MEOX1–ERK1/2–dependent autophagy dysfunction
Source: PLoS Genet. 2026 Oct 8;22(10):e1012329. doi: 10.1371/journal.pgen.1012329 (PMC13648969; doi:10.1371/journal.pgen.1012329)
Supplement: Supplementary file 2 [file pgen.1012329.s001.docx]

**S1 Fig. ZBED6 deficiency leads to the diastolic dysfunction in pig hearts and cardiomyocytes.** (**A**) Western blot analysis of ZBED6 protein levels in hearts from *ZBED6* knockout pigs and their wild type littermates (n = 3). (**B** and **C**) Average fractional shortening and average velocities in cardiomyocytes from WT and *ZBED6*^-/-^ pigs. (**D**) Contraction time, (**E**) relaxation time, (**F**) fractional shortening and (**G**) resting sarcomere length (SL) are shown (N = 3 pigs, n = 50-60 myocytes per group). (**H-M**) Echo parameters of *ZBED6*^−/−^ and WT pigs (n = 4). LVESV: left ventricular end-systolic volume, LVEDV: LV end diastolic volume, LVIDd: LV internal diameter at end diastole, LVIDs: LV internal diameter at end systole, LVPWd: LV posterior wall thickness in diastole, LVPWs: LV end-systolic posterior wall thickness (n = 4). Data are presented as mean ± SEM. **P* < 0.05, ***P* < 0.01.
