# Supplementary material for: ZBED6 deficiency drives pathological cardiac hypertrophy through MEOX1–ERK1/2–dependent autophagy dysfunction
Source: PLoS Genet. 2026 Oct 8;22(10):e1012329. doi: 10.1371/journal.pgen.1012329 (PMC13648969; doi:10.1371/journal.pgen.1012329)
Supplement: Supplementary file 3 [file pgen.1012329.s002.docx]

**S2 Fig. Loss of *ZBED6* upregulates hypertrophic markers in human cardiomyocytes.**

(**A** and **B**) *ZBED6*-KO cardiomyocytes were generated using CRISPR/Cas9, knockout efficiency confirmed by second-generation sequencing and PCR. (**C**) Relative mRNA levels of *ANP*, *BNP* and *MYH7* were analyzed by RT-qPCR in WT and *ZBED6*-deficient AC16 cells treatment with vehicle or Ang II (n = 3). (**D** and **E**) Immunoblotting and quantitation of ANP protein levels in each group (n = 3). Data are expressed as mean ± SEM; * *P* < 0.05, ** *P* < 0.01.
