# Supplementary material for: ZBED6 deficiency drives pathological cardiac hypertrophy through MEOX1–ERK1/2–dependent autophagy dysfunction
Source: PLoS Genet. 2026 Oct 8;22(10):e1012329. doi: 10.1371/journal.pgen.1012329 (PMC13648969; doi:10.1371/journal.pgen.1012329)
Supplement: Supplementary file 4 [file pgen.1012329.s003.docx]

**S3 Fig. *ZBED6* knockout induces mild fibrosis in pigs.** (**A**) Relative mRNA levels of *ANP*, *BNP* and *MYH7* in cardiac tissues from WT and *ZBED6*-deficient pigs (n = 3). (**B**) Western blot analysis of ANP in cardiac tissue in each group (n = 3). (**C and D**) Representative H&E and Masson’s trichrome staining of cardiac tissue from 9-month-old *ZBED6^−/−^* and WT pigs. Scale bar, 50 μm. (**E**) Quantitation of the fibrotic area (%) (n = 3). (**F**) *α-SMA* mRNA expression levels in WT and *ZBED6* knockout pigs (n = 3). Data are expressed as mean ± SEM; * *P* < 0.05, ns, not significant.
