# Supplementary material for: ZBED6 deficiency drives pathological cardiac hypertrophy through MEOX1–ERK1/2–dependent autophagy dysfunction
Source: PLoS Genet. 2026 Oct 8;22(10):e1012329. doi: 10.1371/journal.pgen.1012329 (PMC13648969; doi:10.1371/journal.pgen.1012329)
Supplement: Supplementary file 5 [file pgen.1012329.s004.docx]

**S4 Fig. Mitochondrial structure and function are preserved in ZBED6-Deficient pigs and AC16 cells.** (**A**) Representative electron micrographs showing mitochondrial morphology in left ventricular tissue from WT and *ZBED6*-deficient pigs. Scale bar, 2 μm. (**B**) Protein expression levels of respiratory chain components in cardiac tissues from WT and *ZBED6*-deficient pigs (n = 3). (**C**) Oxygen consumption rate (OCR) in WT and *ZBED6*-deficient AC16 cells (n = 3). (**D** and **E**) The mRNA expression levels of mitochondrial marker genes in *ZBED6*-deficient pig cardiac tissues (n = 4) and *ZBED6*-deficient AC16 cells compared with their control (n = 3). Data are expressed as mean ± SEM; * *P*< 0.05, ** *P* < 0.01, ns, not significant.
