# Supplementary material for: ZBED6 deficiency drives pathological cardiac hypertrophy through MEOX1–ERK1/2–dependent autophagy dysfunction
Source: PLoS Genet. 2026 Oct 8;22(10):e1012329. doi: 10.1371/journal.pgen.1012329 (PMC13648969; doi:10.1371/journal.pgen.1012329)
Supplement: Supplementary file 6 [file pgen.1012329.s005.docx]

 **S5 Fig. ZBED6 deficiency promotes cell apoptosis in pig hearts and human cardiomyocytes.** (A and B) Representative immunofluorescence images of TUNEL staining in hearts from WT and ZBED6-deficient pig (n = 6). Scale bars, 100 μm. (C and D) Representative immunofluorescence images of TUNEL staining in ZBED6-deficient or control AC16 cells (n = 6). Scale bars, 300 μm. Data are expressed as mean ± SEM; * *P*< 0.05, ** *P* < 0.01, ns, not significant.
