# Supplementary material for: ZBED6 deficiency drives pathological cardiac hypertrophy through MEOX1–ERK1/2–dependent autophagy dysfunction
Source: PLoS Genet. 2026 Oct 8;22(10):e1012329. doi: 10.1371/journal.pgen.1012329 (PMC13648969; doi:10.1371/journal.pgen.1012329)
Supplement: Supplementary file 7 [file pgen.1012329.s006.docx]

**S6 Fig. Identification and functional validation of conserved ZBED6 target genes in cardiomyocytes.** (**A**) Bubble plot analysis the 18 genes expression trend in pig and human transcriptome datasets. (**B**) Luciferase reporter assays in WT or *ZBED6*-OE 293T cells transfected with *MEOX1,* *BDNF*, *MCF2L2* and *ARHGAP28* promoter constructs. (**C**) The knockdown efficiency of *BDNF* (left) and *MCF2L2* (right) in AC16 cells treated with si*BDNF* or si*MCF2L2* (n = 3). (**D** and **E**) Representative immunofluorescence images of α-actinin (green) and DAPI (blue) in WT and *ZBED6*-deficient cells transfected with si*BDNF* or si*MCF2L2* for 48 hours. Scale bar, 25 μm. Data are expressed as mean ± SEM; * *P* < 0.05, ** *P* < 0.01, ns, not significant.
