# Supplementary material for: ZBED6 deficiency drives pathological cardiac hypertrophy through MEOX1–ERK1/2–dependent autophagy dysfunction
Source: PLoS Genet. 2026 Oct 8;22(10):e1012329. doi: 10.1371/journal.pgen.1012329 (PMC13648969; doi:10.1371/journal.pgen.1012329)
Supplement: Supplementary file 8 [file pgen.1012329.s007.docx]

 **S7 Fig. ZBED6 controls the transcription of a cardiac hypertrophy regulator MEOX1.** (**A**) Relative mRNA expression level of *MEOX1* in WT and *ZBED6*-depletion cardiomyocytes (n = 3). (**B** and **C**) The protein level of MEOX1 in *ZBED6*-knockout porcine hearts and human cardiomyocytes compared with controls (n = 3). (**D**) MEOX1 protein level in cardiac tissue from healthy individuals (n = 6) and cardiac hypertrophy patients (n = 5). (**E**) Schematic of wild-type and ZBS-mutant sequences. (**F**) Luciferase activity of wild-type (ZBS-LUC) or mutant (mZBS-LUC) *MEOX1* reporters in the presence of ZBED6 (n = 6). (**G**) ChIP assay analysis of ZBED6 binding to the *MEOX1* promoter region in pig cardiac tissue (n = 3). (**H**) The relative luciferase activity of the *MEOX1*-ZBS-LUC reporter in *ZBED6*-deficient and control cells (n = 12). Data are expressed as mean ± SEM; * *P* < 0.05, ** *P* < 0.01.
