# Supplementary material for: ZBED6 deficiency drives pathological cardiac hypertrophy through MEOX1–ERK1/2–dependent autophagy dysfunction
Source: PLoS Genet. 2026 Oct 8;22(10):e1012329. doi: 10.1371/journal.pgen.1012329 (PMC13648969; doi:10.1371/journal.pgen.1012329)
Supplement: Supplementary file 9 [file pgen.1012329.s008.docx]

 **S8 Fig. ZBED6 deficiency-induced cardiomyocyte hypertrophy is independent of IGF2.** (**A**) Relative *IGF2* mRNA levels in each group (n = 3). (**B**) The protein levels of IGF2, p-ERK1/2, and total ERK1/2 in each group (n = 3). (**C**) The quantization of IGF2 protein levels in each group (n = 3). (**D** and **E**) Representative immunofluorescence images of α-actinin and quantification of cell size in ZBED6-deficient AC16 cells treated with si-*IGF2* or si-*Control* for 48 hours (n = 50). Scale bars, 25 μm. Data are presented as mean ± SEM. **P* < 0.05, ***P* < 0.01, ns, not significant.
