# Supplementary material for: ZBED6 deficiency drives pathological cardiac hypertrophy through MEOX1–ERK1/2–dependent autophagy dysfunction
Source: PLoS Genet. 2026 Oct 8;22(10):e1012329. doi: 10.1371/journal.pgen.1012329 (PMC13648969; doi:10.1371/journal.pgen.1012329)
Supplement: Supplementary file 10 [file pgen.1012329.s010.docx]

**S1 Table**

| **Species** | **Genes** | **Primers** | **Size (bp)** | **Accession Number** |
| --- | --- | --- | --- | --- |
| Human | *NPPA* | F: CTAAAAAGCAAGCTGAGGGCG | 188 | NM_006172.4 |
|  |  | R: ACAGGAGCCTCTTGCAGTCT |  |  |
|  | *NPPB* | F: TGGAAACGTCCGGGTTACAG | 122 | NM_002521.3 |
|  |  | R: CTTCCAGACACCTGTGGGAC |  |  |
|  | *MYH7* | F: GAGGAGCAAGCCAACACCAA | 151 | NM_000257.4 |
|  |  | R: ACTCCTCATTCAAGCCCTTCG |  |  |
|  | *MEOX1* | F: TGACTCGGCTCCGCAGATATG | 102 | NM_001040002.2 |
|  |  | R: TCACACGCTTCCACTTCATCCT |  |  |
|  | *ND1* | F: TCCTGCCATCATGACCCTTG | 129 | NC_012920.1 |
|  |  | R: CTGCGGCGTATTCGATGTTG |  |  |
|  | *ND2* | F: CAGAGGTTACCCAAGGCACC | 160 | NC_012920.1 |
|  |  | R: CCACCTCAACTGCCTGCTAT |  |  |
|  | *ND3* | F: GCGGCTTCGACCCTATATCC | 121 | NC_012920.1 |
|  |  | R: AGGGCTCATGGTAGGGGTAA |  |  |
|  | *CYTB* | F: CCGCATGATGAAACTTCGGC | 132 | NC_012920.1 |
|  |  | R: CTCGAGTGATGTGGGCGATT |  |  |
|  | *COX1* | F: TCGGAGGCTTTGGCAACTGACT | 124 | NC_012920.1 |
|  |  | R: GCAGATGCGAGCAGGAGTAGGA |  |  |
|  | *COX2* | F: CTATCCTGCCCGCCATCATCCT | 147 | NC_012920.1 |
|  |  | R: CGCCGTAGTCGGTGTACTCGTA |  |  |
|  | *COX3* | F: ACCCTCCTACAAGCCTCAGAGT | 102 | NC_012920.1 |
|  |  | R: GTGAAGTCCGTGGAAGCCTGTG |  |  |
|  | *ATP6* | F: CTCGGACTCCTGCCTCACTCAT | 155 | NC_012920.1 |
|  |  | R: GGTGTAGGTGTGCCTTGTGGTA |  |  |
|  | *GAPDH* | F: ACCCACTCCTCCACCTTTGAC | 100 | NM_001289745.3 |
|  |  | R: TGTTGCTGTAGCCAAATTCGTT |  |  |
| Pig | *GAPDH* | F:CCTTCATTGACCTCCACTACATGG | 212 | NM_001206359.1 |
|  |  | R: CTCCATGGTCGTGAAGACACC |  |  |
|  | *NPPA* | F: GACCACTTGGAGGACAAGATG | 74 | NM_214260.2 |
|  |  | R: TCCTCATTCTGCTCGCTTAGT |  |  |
|  | *NPPB* | F: CGCAGTAGCATCTTCCAAGTC | 130 | NM_213846.1 |
|  |  | R: ACCTCCTGAGCACATTGCAG |  |  |
|  | *MYH7* | F: ACACCAACCTGTCCAAGTTCC | 162 | NM_213855.2. |
|  |  | R: GGCAGATCAAGATGTGGCAAA |  |  |
|  | *ACTA2* | F: CTGCCCTGGTGTGTGACAAT | 92 | NM_001164650.1 |
|  |  | R: TCCCACAATGGACGGGAAAA |  |  |
|  | *ND2* | F: ACGACCAGGCACATCCTCAATC | 158 | NC_000845.1 |
|  |  | R: AGACTGCTAGGCTTGACATGGC |  |  |
|  | *ND3* | F: CAGCACGCCTCCCATTCTCAAT | 173 | NC_000845.1 |
|  |  | R: TGCTAGGCTTGCTGCTAGTAGG |  |  |
|  | *ND6* | F: TATACTACTGCTATGGCTACTG | 134 | NC_000845.1 |
|  |  | R: TCTACTTCCTCTTCCTTCAAC |  |  |
|  | *COX1* | F: GGAGCGGGTACTGGATGAAC | 103 | NC_000845.1 |
|  |  | R: CACCTGCAAGGTGTAGGGAG |  |  |
|  | *COX2* | F: GCTTCCAAGACGCCACTTCACC | 154 | NC_000845.1 |
|  |  | R: TGGGCATCCATTGTGCTAGTGT |  |  |
|  | *COX3* | F: CCTAGCACCAACACCCGAAT | 113 | NC_000845.1 |
|  |  | R: ATACTCCTGAGGCGAGGAGG |  |  |
|  | *ATP6* | F: ACCGCACAATCTCGATCCAACA | 170 | NC_000845.1 |
|  |  | R: TGTGGTGGGTGTGAATGAGTGT |  |  |
|  | *BDNF* | F: AACCGGGCACCAAAGATTCC | 171 | NM_214259.2 |
|  |  | R: GGTCATCACTCTTCTCACCTGG |  |  |
|  | *MCF2L2* | F: TGTTGGCAGAGCAAGCAGAGTG | 193 | XM_021069898.1 |
|  |  | R: GCACACCTGGGTCTGATGGAGT |  |  |
|  | *MEOX1* | F: GTGCCAATGAGACGGAGAAGAA | 100 | XM_005668770 |
|  |  | R: CCTTCACACGCTTCCACTTCAT |  |  |
| ChIP-PCR | | F: AATAGTTGCCTGCTCGGGTTT | 140 | NA |
|  |  | R: AGGGCTGCTGAGACACACTTT |  |  |
