# Supplementary material for: ZBED6 deficiency drives pathological cardiac hypertrophy through MEOX1–ERK1/2–dependent autophagy dysfunction
Source: PLoS Genet. 2026 Oct 8;22(10):e1012329. doi: 10.1371/journal.pgen.1012329 (PMC13648969; doi:10.1371/journal.pgen.1012329)
Supplement: Supplementary file 11 [file pgen.1012329.s011.docx]

**S2 Table**

| **Antibody** | **Source** | **Product Number** | **Dilution (WB/IF/IP)** |
| --- | --- | --- | --- |
| ANP | Wanlei | WL02666 | 1:1000/no/no |
| BNP | Santa | sc-271185 | 1:500/no/no |
| LC3B | Immunoway | YM8147 | 1:1000/no/no |
| P62 | Immunoway | YM8025 | 1:1000/no/no |
| ZBED6 | BT Lab | BT-AP13622 | 1:1000/no/no |
| ERK1/2 | Vazyme | Ra2201 | 1:1000/no/no |
| p-ERK1/2 | Vazyme | Ra2202 | 1:1000/no/no |
| P38 | lmmunoway | YT3513 | 1:1000/no/no |
| p-P38 | Wanlei | WLP1576 | 1:1000/no/no |
| JNK1/2/3 | Immunoway | YT2440 | 1:1000/no/no |
| p-JNK1/2/3 | Immunoway | YP0157 | 1:1000/no/no |
| MEOX1 | Proteintech | 18190-1-AP | 1:500/no/no |
| GAPDH | Proteintech | 60004-1-lg | 1:5000/no/no |
| Total OXPHOS | Abcam | ab110413 | 1:1000/no/no |
