# Supplementary material for: ZBED6 deficiency drives pathological cardiac hypertrophy through MEOX1–ERK1/2–dependent autophagy dysfunction
Source: PLoS Genet. 2026 Oct 8;22(10):e1012329. doi: 10.1371/journal.pgen.1012329 (PMC13648969; doi:10.1371/journal.pgen.1012329)
Supplement: Supplementary file 12 [file pgen.1012329.s012.docx]

**S3 Table. siRNA sequences.**

| **Targeting gene** | **RNA Oligo sequence** | |
| --- | --- | --- |
| Human *MEOX1* | SS: CGUUCUCCUUCCACCAGAAAC | AS: UUCUGGUGGAAGGAGAACGGG |
| Human *BDNF* | SS: UGAUGCUCAGUAGUCAAGU | AS: UUGACUACUGAGCAUCACCCU |
| Human *MCF2L2* | SS: AGAACAACUUCACAGACAAUU | AS: UUGUCUGUGAAGUUGUUCUAU |
